# Deletion of *Vhl* in *Dmp1*-Expressing Cells Causes Microenvironmental Impairment of B Cell Lymphopoiesis

**DOI:** 10.3389/fimmu.2022.780945

**Published:** 2022-02-16

**Authors:** Betsabel Chicana, Nastaran Abbasizadeh, Christian Burns, Hanna Taglinao, Joel A. Spencer, Jennifer O. Manilay

**Affiliations:** ^1^ Department of Molecular and Cell Biology, School of Natural Sciences, University of California, Merced, Merced, CA, United States; ^2^ Quantitative and Systems Biology Graduate Program, University of California, Merced, Merced, CA, United States; ^3^ Department of Bioengineering, School of Engineering, University of California, Merced, Merced, CA, United States; ^4^ Bioengineering Graduate Program, University of California, Merced, Merced, CA, United States

**Keywords:** B lymphocytes, osteoimmunology, hypoxia, microenvironment, bone marrow niches

## Abstract

The contributions of skeletal cells to the processes of B cell development in the bone marrow (BM) have not been completely described. The von-Hippel Lindau protein (VHL) plays a key role in cellular responses to hypoxia. Previous work showed that *Dmp1*-Cre;*Vhl* conditional knockout mice (*Vhl*cKO), which deletes *Vhl* in subsets of mesenchymal stem cells, late osteoblasts and osteocytes, display dysregulated bone growth and reduction in B cells. Here, we investigated the mechanisms underlying the B cell defects using flow cytometry and high-resolution imaging. In the *Vhl*cKO BM, B cell progenitors were increased in frequency and number, whereas Hardy Fractions B-F were decreased. *Vhl*cKO Fractions B-C cells showed increased apoptosis and quiescence. Reciprocal BM chimeras confirmed a B cell-extrinsic source of the *Vhl*cKO B cell defects. In support of this, *Vhl*cKO BM supernatant contained reduced CXCL12 and elevated EPO levels. Intravital and *ex vivo* imaging revealed *Vhl*cKO BM blood vessels with increased diameter, volume, and a diminished blood-BM barrier. Staining of *Vhl*cKO B cells with an intracellular hypoxic marker indicated the natural existence of distinct B cell microenvironments that differ in local oxygen tensions and that the B cell developmental defects in *Vhl*cKO BM are not initiated by hypoxia. Our studies identify novel mechanisms linking altered bone homeostasis with drastic BM microenvironmental changes that dysregulate B cell development.

## Introduction

The mechanisms by which changes in bone homeostasis affect immune development in the bone marrow (BM) are not fully understood (1–4). A detailed understanding of how bone microenvironments affect immune cell development and function could provide strategies towards novel therapeutic approaches to immune deficiencies. B cells produce antibodies (Abs), which are crucial for a robust adaptive immune response. B cells are generated from hematopoietic stem cells (HSCs) in the liver during fetal life, and in the BM in the adult (5). B cell development in the BM occurs in a series of defined stages that rely on growth factors that are produced by several non-hematopoietic stromal cells, including mesenchymal stem cells (MSCs) and osteoblasts (OBs) (1).

The von-Hippel Lindau protein (VHL) regulates hypoxia-inducible factor (HIF) degradation, which is involved in cellular adaptation to low oxygen environments (6). When HIF1α accumulates in normoxic conditions, it travels to the nucleus to activate over 100 hypoxia-inducible target genes (7). VHL is expressed ubiquitously in many cell types, and global deletion of the *Vhl* gene results in embryonic lethality, so conditional knockout approaches are necessary to investigate the cell-specific roles of VHL in specific microenvironments. Conditional deletion of *Vhl* in OBs and in hematopoietic progenitors have demonstrated a role for VHL in these cell types (8, 9). The role of HIF and its regulation on the immune system has been extensively reviewed (10), but the mechanisms by which cell-intrinsic and cell-extrinsic VHL regulate specific immune cell lineages has not fully been addressed.

The BM microenvironment manifests hypoxic heterogeneities in a spatio-temporal manner (11–13), however the implications of these oxygen tension (pO_2_) differences on hematopoiesis are not well characterized. Hypoxia slows the processes of angiogenesis and osteogenesis during fracture healing and bone formation, but also promotes OB differentiation into OCYs (14), and can stimulate osteoclast formation (15). Studies have shown HIF stabilization as a therapeutic option for treating bone fractures (16, 17) and osteoporosis (18–20), but the underlying molecular mechanism remains poorly understood. *Vhl* plays an important role regulating HIF expression, and disruption of *Vhl* in bone cells leads to improper bone homeostasis (7, 8, 21, 22). *Vhl* depletion in osteochondral progenitor cells and osteocalcin-positive OBs leads to an increase in bone mass through an increase in OB number (7, 22). Furthermore, disrupting VHL in OBs induces expression of β-catenin, revealing the mechanism by which VHL/HIF pathway promotes bone formation through the Wnt pathway (7, 23, 24). Altogether, these studies of *Vhl* deletion in osteolineage cells have not examined the cell-extrinsic effects of these changes on the immune cells residing in the BM.

The BM contains specialized microenvironments that maintain blood cells and supply factors required for their development and maintenance. Perivascular stromal cells, osteoprogenitor cells, endothelial cells (ECs), MSCs, OBs and OCYs are critical B cell “niches” and are all cells that support B cell development (1, 4, 25, 26), in part through production of cytokines. Essential cytokines for B cell development include CXC-chemokine ligand 12 (CXCL12) (27–29), FLT3 ligand (FLT3L) (30), IL-7 (30–33), stem-cell factor (SCF) (31, 32) and receptor activator of nuclear factor-κB ligand (RANKL) (34). The BM contains a dense vascular network and vascular sinuses creating the perivascular region, which provides a niche where B cells are known to develop and reside (35). A model of B cell developmental niches based on CXCL12 and IL7 levels has been proposed (4) in which B cells start at the pre-pro-B cell (Fraction A) stage where they are located in the perisinusoidal niche, especially near CXCL12+ reticular cells. As B cells continue to mature to the pro-B cell stage (Fractions B-C), they also interact with IL-7 expressing cells, and then pre-B cells migrate away from the sinusoids toward galectin-1+ stromal cells that do not express IL7 (36). This model has been updated given recent reports of the four new MSC subsets, their ability to support B lymphopoiesis, and their locations within the BM (37). During aging, vascular density decreases in many tissues due to impaired angiogenesis caused by EC dysfunction (38, 39). Vascular “hyperpermeability” also increases with age, via changes in ECs lining the blood vessel wall, disrupting the blood-BM barrier (40–42). The role of the vasculature and regulation of vessel permeability in hematopoiesis, especially in B cell development, remains unknown.

To understand how changes in bone homeostasis may affect immune cell development, we previously utilized *Dmp1*-Cre;*Vhl* conditional knockout mice (*Vhl*cKO), in which *Vhl* is deleted primarily in OCYs, but also in some MSC subsets and late OBs (43). In the *Vhl*cKO bones, the number of hematopoietic cells is severely reduced, and B cell development is stunted (21). Here, we provide evidence for molecular, cellular and structural changes in the *Vhl*cKO BM niche that adversely affect B cell development in a cell-extrinsic manner, such as decreased production of B cell supporting cytokines and structural changes in the BM vasculature. We also observed an age-dependent change in hypoxia that could further contribute to the B cell defects. These studies reveal novel molecular mechanisms by which *Vhl* deletion in *Dmp1*-expressing cells affect B cell niches.

## Materials and Methods

### Study Design

A G*Power statistical (44) power analysis (α=0.05 and power of 0.95) based on B cell developmental data and BM cellularity determined that a minimum of n=7 mice per group was needed for our studies. The total sample size for each experiment was >7 performed in three independent experiments. Age-matched mice of both sexes were used. *Vhl*cKO and control mice (C57BL/6 wild type and *Vhl*-floxed (*Vhl^fl/fl^*, *Dmp1-Cre-*negative mice) were used and no sex-specific differences in B cell development or other relevant characteristics to our studies were detected. Student’s t-test and nonparametric Bonferroni-corrected Mann-Whitney U-test was used to test differences between mean and median values with Graph-Pad Prism and were considered significant if p<0.05. Outlier analysis was also performed with Graph-Pad Prism and any outliers identified were not included in the data graphs.

### Experimental Animals

Mice on the C57Bl/6 background were used. Stock #023047 B6N.FVB-Tg1Jqfe/BwdJ (*Dmp1*-Cre) (45) and Stock #012933 B6.129S4(C)-Vhl tm1Jae/J (*Vhl^fl/fl^*) (46) were purchased from The Jackson Laboratory. These two lines of mice were crossed to generate *Vhl* conditional knockouts in *Dmp1*-expressing cells (*Vhl*cKO). Genotyping was confirmed following protocols from the Jackson Laboratory. Stock #002014 B6.SJL-Ptprca Pepcb/BoyJ mice were used for reciprocal bone marrow transplantation studies. Mice were housed under specific pathogen-free conditions in the University of California, Merced’s vivarium with autoclaved feed and water, and sterile microisolator cages. The University of California Merced Institutional Animal Care and Use Committee approved all animal work.

### Bone Marrow Transplantation

Recipient mice were 10 weeks of age at the time of transplantation. Whole bone marrow B6.SJL (CD45.1+) donor cells (1x10^6^) were injected retro-orbitally into lethally irradiated (1000 rads using a Cesium-137 source, JL Shepherd and Associates, San Fernando, CA, USA) recipient CD45.2+ *Vhl*cKO mice or control (Cre-negative; *Vhl^fl/fl^*) littermates under isoflurane anesthesia. Reciprocal *Vhl*cKO→WT (B6.SJL, CD45.1+) chimeras were also prepared. Animals were supplemented with neomycin in the drinking water for 14 days post-transplant as described (47).

### Sample Collection: Bone Marrow, Peripheral Blood, Spleen and Serum

#### Bone Marrow Collection

Mice were euthanized by the inhalation of carbon dioxide followed by cervical dislocation. Femurs and tibias were dissected, and muscles were removed. To release the BM, bones were crushed with a mortar and pestle in M199+ (M199 with 2% FBS). BM cells were collected into 15mL conical tubes after being rinsed away from bone chips with M199+, resuspended by trituration, filtered through 70-micron nylon mesh into a 50 mL conical tube, and centrifuged for 5 mins at 1500 rpm and at 4°C. Cell pellets were resuspended and treated with ACK lysis buffer to remove erythrocytes. Cells treated with ACK were washed and resuspended in M199+. Cell counts were obtained using a hemocytometer and Trypan Blue staining to exclude dead cells.

To collect BM supernatant, femurs were cleaned of any muscle tissue and the epiphyses were cut off and discarded. The bone shaft was then placed into a 0.2 mL tube in which a hole was introduced using a needle. Thirty µL of 1x phosphate buffered saline (PBS) was placed on the top end of the bone shaft, using a 25g needle, and then the tube containing the bone was placed into a 1.5 ml microcentrifuge tube and centrifuged for 30 seconds at 15,000rpm. The BM supernatant was collected and stored at -80C until analysis.

#### Peripheral Blood Collection

Mice were heated under a heat lamp to increase blood circulation and then restrained. Blood collection was performed via tail bleeds by making an incision with a scalpel blade over the ventral tail vein. No more than ten drops were collected (<0.5 mL) in a 1.5 ml Eppendorf tube with 50 uL of heparin. To obtain blood serum, blood was collected in 1.5 ml tubes without heparin and allowed to clot for 30 minutes at room temperature. The samples were then centrifuged for 10 minutes at 4000 rpm at 4°C. Blood serum was collected and stored at -80°C until the day of analysis.

#### Spleen Cell Collection

Dissected spleens were processed and mashed in 1 mL of ACK lysis buffer in a petri dish for no more than one minute. Five mL of M199+ were added into the dish to dilute the ACK lysis buffer and to stop red cell lysis. Spleen cells were aspirated into a 5mL syringe to create single cell suspensions by passing the cells through the syringe several times then filtering through a 70-micron nylon mesh into a 15 mL conical tube. Cells were centrifuged at 2000 rpm at 4°C for 3 minutes. Cell pellets were loosened by gently tapping the tubes by hand before resuspending the cells in 5 mL of M199+. Live cell counts were determined using a hemocytometer and Trypan Blue staining.

### Quantification of Cytokines

Cytokine measurements were performed using a customized bead-based multiplex (13-LEGENDplex assay) from Biolegend, Inc. with the analytes IL-3, IL-5, IL-6, IL-7, IL-15, IL-34, M-CSF, TPO, GM-CSF, LIF, EPO, CXCL12, SCF for the analysis of BM serum and peripheral blood serum of *Vhl*cKO and control mice. Concentrations of cytokines were determined from samples following manufacturer’s instructions and software.

### Flow Cytometry Analysis and Antibodies

Cells were stained for flow cytometry and included a pre-incubation step with unconjugated anti-CD16/32 (clone 93) to block Fc receptors as previously described (47, 48). The antibody cocktails used for different sets of stains are listed in **Supplementary Table 1**. For viability staining, DAPI (Sigma-Aldrich, 0.005 μg/ml final concentration) or propidium iodide (Sigma-Aldrich, 0.025 μg/ml final concentration) was used. Single color stains were used for setting compensations and gates were determined with fluorescent-minus one controls, isotype-matched antibody controls, or historical controls. Intracellular staining of Ki67 was performed using the eBioscience™ Foxp3/Transcription Factor Staining Buffer Set following the manufacturer’s instructions. For cell cycle analysis, DAPI was used at a final concentration of 0.1 μg/ml per sample. Apoptosis staining was performed using Biolegend Annexin V Apoptosis Detection Kit with 7AAD. Flow cytometry data was acquired on the BD LSR II. The data was analyzed using FlowJo Software version 10.7.1.

### Preparation of Long Bones for Imaging

To label blood vessels, mice were injected with fluorescent antibodies (**Supplementary Table 1**) through the retro-orbital venous sinus. After 20 minutes of incubation, intracardial perfusion was performed with 1X PBS following by cold and fresh 4% paraformaldehyde (PFA). Subsequently, femurs were harvested and fixed in the 4% PFA for 30 minutes, at 4°C. The bones were then washed with 1X PBS, immersed in 30% sucrose for 1 hour, frozen in optimal cutting temperature (OCT) compound and kept at – 80°C. Samples were shaved using a cryostat (LEICA CM1860) equipped with a high-profile blade (Leica; 3802121).

To optically clear long bones, a modified uDISCO clearing protocol was used (49). After intracardial perfusion as described above, long bones were immersed in 4% PFA overnight and put through a series of *tert*-butanol (Sigma-Aldrich, SHBM5332) dehydration steps at 30% (4 hours), 50% (4 hours), 70% (overnight), 80% (4 hours), 90% (4 hours), and 100% (overnight). Next, long bones were incubated in dichloromethane (DCM; Sigma-Aldrich, SHBJ8352) for 40 minutes and then placed in Benzyl Alcohol (Sigma-Aldrich, SHBK5469) Benzyl Benzoate (Sigma-Aldrich, MKCM1445) - DL-alpha-tocopherol (Alfa Aesar, Y04D032) (BABB-D4) for 3-4 hours. BABB-D4 is prepared by mixing Benzyl Alcohol + Benzyl Benzoate at the ratio of 1:2, adding diphenyl ether (DPE; Sigma-Aldrich, SHBL5909) to the BABB solution (1:4) and ultimately DL-alpha-tocopherol (Vitamin E) with the ratio of 1:25 to decrease fluorescence quenching. Cleared femurs were mounted in a custom glass chamber filled with BABB-D4 and sealed with solvent-resistant silicone gel (DOWSIL™ 730) (49).

### Two-Photon Microscopy

Imaging was performed with a custom-built two-photon video-rate microscope (Bliq Photonics) equipped with two femtosecond lasers (Spectra Physics; Insight X3, Spectra Physics; MaiTai eHP DS). During intravital imaging, the Spectra Physics Insight X3 and Maitai laser wavelengths were tuned to 840 nm and 1040 nm, respectively, and for *ex vivo* imaging only the Insight X3 was tuned to 1220 nm. Three fluorescent channels were acquired (503-538 nm, 572-608 nm, and 659-700 nm). For all two-photon imaging, a 25x water immersion objective (Olympus; XLPLN25XWMP2) with 1.05 numerical aperture was used to image a 317 μm by 159 μm field of view. Videos were recorded at 30 frames per second and images were generated by averaging of 30 frames from the live video mode.

For *in vivo* imaging of calvarial bone marrow, mice were anesthetized with isofluorane (3-4% induction, 1.5% maintenance at 1L/min) and the top of the head shaved. The skin was cleaned with 70% alcohol wipes before surgery. The mouse was placed on a heating pad and secured in a custom head mount. An incision was made along the sagittal and lambda suture of the skull and the skin retracted to expose the calvarial bone as previously described (11, 50). The secured mouse was then placed on the microscope stage for two-photon microscopy (11, 50). In order to measure BM blood vessel permeability, leakage and flow velocity in the calvaria BM during *in vivo* imaging, 70 kDa Rhodamine-B-Dextran (ThermoFisher, D1841) was injected retro-orbitally while the mouse was on the stage.

For *ex vivo* imaging, optically cleared long bones were mounted in a chamber sealed with solvent-resistant silicone gel (DOWSIL™ 730) and shaved long bones were mounted on a wet sponge to prevent the sample from drying during imaging. Slides were imaged with similar acquisitions settings as the *in vivo* imaging.

### Image Quantification

For *in vivo* image analysis, image processing and permeability/leakage measurements were performed with Fiji (ImageJ 1.53k) and BM blood flow velocity was quantified with custom scripts in MATLAB (2020a). To measure permeability in the calvaria, live two-photon microscopy video was recorded for the first 30 seconds after Rhodamine B Dextran was injected. The blood vessel permeability was calculated based on the change in fluorescence intensity outside of blood vessels over time as previously described (51, 52). For leakage measurements, z-stacks (2 µm step size) were recorded randomly around the calvarium BM 10 minutes after injection. Leakage values were calculated by dividing the fluorescence intensity of the perivascular space adjacent to a vessel by the fluorescence intensity inside the blood vessel. Representative examples of BM leakage were generated by taking maximum intensity projections (MIPs) of BM regions with image contrast/enhancement applied. Blood flow velocity was calculated by recording 30 second videos of blood flow in the BM calvaria and then utilizing the Line Scanning Particle Image Velocimetry (LSPIV) method implemented in a custom MATLAB script to calculate blood flow velocity as previously described (53, 54). ImageJ (ImageJ 1.53k) was used to adjust video and image contrast for figure presentation.

In long bone images, as required, 3D z-stacks were rotated with the “TransformJ” plugin in ImageJ to exclude the non-relevant signals and final images were generated by taking maximum intensity projections (MIPs) of BM regions and adjusting the image contrast/enhancement. To generate a depth-dependent profile of vessel diameter in long bones, measurements were taken at 0-30 μm (shallow BM), 75-105 μm (middle BM), and 150-180 μm (deep BM) below the endosteum. To measure vascular density, image brightness/contrast was first adjusted in Fiji (ImageJ 1.53k) and then images were converted to binary. Next, noise reduction was performed *via* Despeckle, and binary Fill Hole was applied. Finally, using analytical coding developed in Python (3.7.6), the ratio of the total blood vessel pixels to total BM pixels was determined for BM vessel density measurements.

## Results

### *Vhl* Deletion in Dmp1-Expressing Cells Dysregulates Hematopoiesis

**Figure 1:**
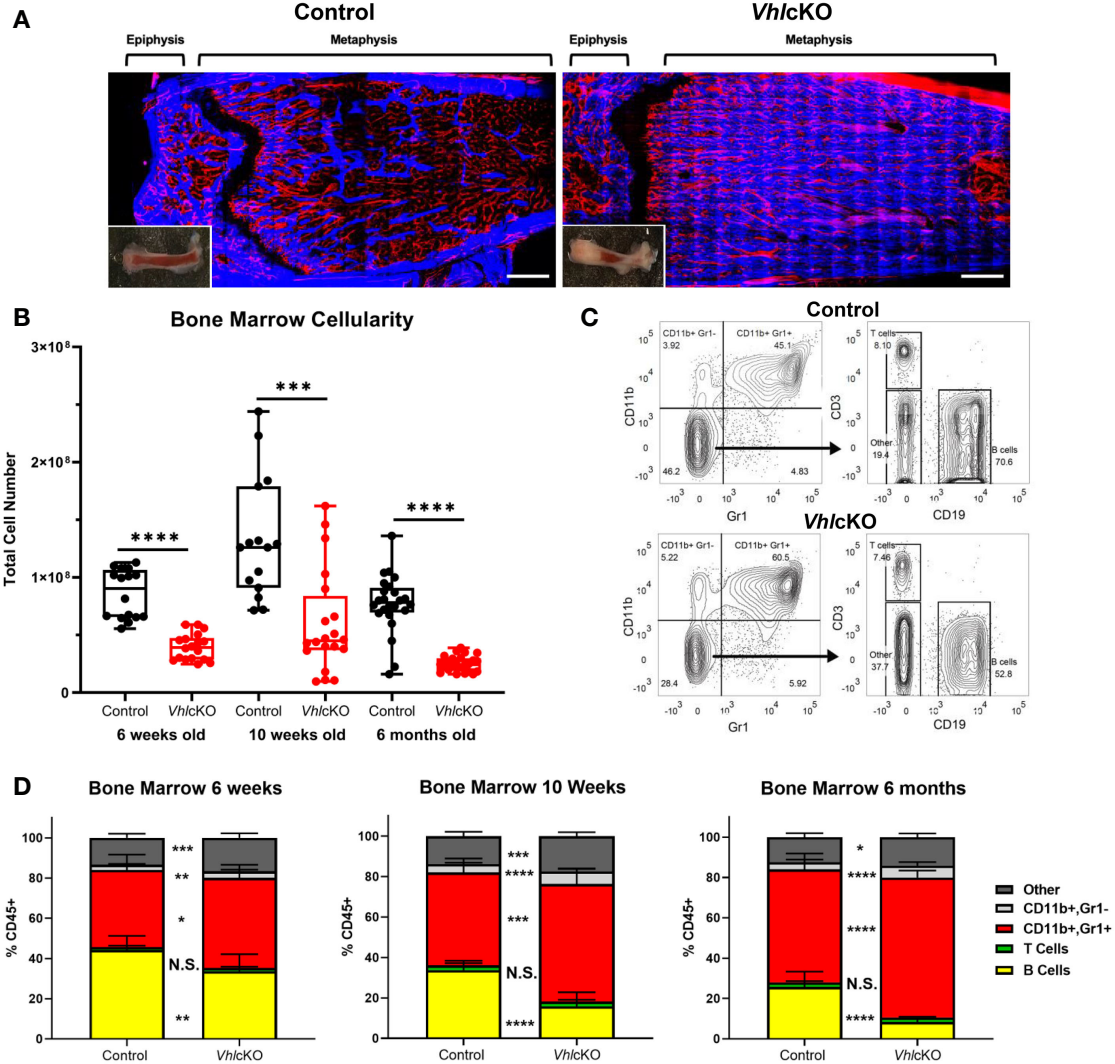
Bone marrow, spleen and peripheral blood lineage cell defects in the *Vhl*cKO mice. **(A)** Macroscopic and *ex vivo* imaging of the distal end of long bones revealed progressive increases in the bone mass of 10-weeks-old *Vhl*cKO femurs compared to control. Inset: photo of the femur. Red: blood vessel (AlexaFluor647 CD31, AlexaFluor647 CD144, AlexaFluor647 Sca-1), Blue: bone (SHG). Scale bar ~500μm; **(B)** bone marrow cellularity, **(C)** representative FACS plots of immune cell lineages, with the values on plots on the left representing cell frequency in total bone marrow, and the values on the plots on the right representing frequencies within the Gr-1- CD11b- gate. **(D)** frequency analysis of bone marrow lineage cells at 6-weeks of age (left), 10-weeks of age (middle) and 6-month (right). p<0.05*, p<0.01**, p<0.001***, p<0.0001**** two-tailed Student’s t-test. N.S., not statistically significant.

Previous studies of *Vhlc*KO mice utilized mice on a mixed genetic background (21). For our studies, we required a pure C57BL/6 (B6) background and we performed a thorough comparison of our B6 *Vhl*cKO mice to previous published results. Similar to previous reports (21), we found that long bones in B6 *Vhl*c*KO* mice display abnormally high bone mass and density and the BM cavity is severely occluded with bone (**Figure 1A**), accompanied by stunted B cell development, splenomegaly (**Supplementary Figures 1A–E**), and reduced BM cellularity compared to controls (**Figure 1B**). In the B6 *Vhl*cKO, we extended our analysis to be longitudinal, examining hematopoietic lineages at multiple ages. Analysis of specific hematopoietic cell lineages in the BM revealed a decrease in B cells, no change in T cell frequency, and an increase in CD11b+ Gr1- cells (enriched for monocytes) and CD11b+ Gr1+ cells (enriched for Ly6G+ granulocytes, but also may include CD115+ and Ly6C+ monocytes) in 6-week-old, 10-week-old and 6-month-old mice (**Figures 1C, D**). Furthermore, an overall reduction in the absolute numbers of all hematopoietic lineages in the BM of *Vhlc*KO mice was observed (**Table 1**). Lineage analysis in the spleen at 10 weeks revealed a decrease in B cells, no change in T cells, and an increase in CD11b+ Gr1+ cells that became more prominent as mice aged to 6 months. CD11b+ Gr1- cells in the *Vhl*cKO spleen at 6-weeks-old were slightly reduced, similar to controls at 10-weeks-old, and were increased at 6-months-old (**Supplementary Figure 1E**). Peripheral blood of the *Vhl*cKO mice showed no change in B cells at 6 weeks, but B cells were decreased at 10 weeks and 6 months. In contrast, CD11b+ Gr1- cells were increased at 10-weeks-old, and CD11b+ Gr1+ cells at 6-months-old only (**Supplementary Figure 1F**).

**Table 1:** Hematopoetic lineage mean±SD and absolute number p<0.05*, p<0.01**, p<0.001***, p<0.0001**** two-tailed Student’s t-test.

	CD45+ Population (mean% ± SD)				Absolute Number (mean ± SD)			
Lineage Population	Bone Marrow		Spleen		Bone Marrow		Spleen	
6 weeks old	Control	*Vhl* cKO	Control	*Vhl* cKO	Control	*Vhl* cKO	Control	*Vhl* cKO
B cells	44.16 ± 7.16	33.69 ± 8.49**	60.71 ± 2.64	58.57 ± 3.51	2.62E+0.7 ± 7.06E+06	8.88E+0.6 ± 4.83E+06****	6.35E+0.7 ± 1.28E+07	5.71E+0.7 ± 9.18E+06
T cells	1.45 ± 0.79	1.61 ± 0.62	23.65 ± 3.56	23.49 ± 3.42	8.36E+0.5 ± 4.33E+05	3.70E+0.5 ± 1.87E+05***	2.49E+0.7 ± 6.57E+06	2.29E+0.7 ± 4.93E+06
CD11b+ Gr1-	2.58 ± 0.37	3.28 ± 0.74**	2.55 ± 0.57	1.95 ± 0.15**	1.53E+0.6 ± 3.92E+05	7.81E+0.5 ± 3.48E+05****	2.66E+0.6 ± 7.87E+05	1.90E+0.6 ± 2.96E+05*
CD11b+ Gr1+	38.46 ± 7.61	44.85 ± 6.47*	2.85 ± 2.55	2.28 ± 0.66	2.42E+0.7 ± 1.12E+07	1.06E+0.7 ± 4.30E+06***	2.71E+0.6 ± 2.19E+06	2.17E+0.6 ± 5.06E+05
**10 weeks old**	Control	*Vhl* cKO	Control	*Vhl* cKO	Control	*Vhl* cKO	Control	*Vhl* cKO
B cells	33.8 ± 4.55	15.83 ± 7.01****	60.71 ± 7.63	46.37 ± 7.53****	3.32E+0.7 ± 1.47E+07	8.02E+0.6 ± 1.29E+07***	1.22E+08 ± 6.02E+07	1.18E+08 ± 5.98E+07
T cells	2.34 ± 1.04	2.35 ± 0.95	26.67 ± 6.15	29.09 ± 4.11	2.55E+0.6 ± 1.92E+06	1.05E+0.6 ± 1.35E+06*	6.22E+07 ± 5.28E+07	8.10E+07 ± 5.40E+07
CD11b+ Gr1-	4.09 ± 0.67	6.23 ± 1.25****	2.50 ± 0.36	2.71 ± 0.38	4.08E+0.6 ± 2.08E+06	2.41E+0.6 ± 1.89E+06*	5.49E+06 ± 4.12E+06	7.02E+06 ± 3.81E+06
CD11b+ Gr1+	45.93 ± 6.89	58.11 ± 7.78***	1.15 ± 0.50	6.27 ± 2.93****	4.36E+0.7 ± 1.60E+07	2.22E+0.7 ± 1.50E+07**	2.14E+06 ± 9.96E+05	1.66E+07 ± 1.46E+07**
**6 months old**	Control	*Vhl* cKO	Control	*Vhl* cKO	Control	*Vhl* cKO	Control	*Vhl* cKO
B cells	25.74 ± 7.62	8.37 ± 1.76****	61.38 ± 7.09	42.44 ± 3.65****	1.48E+0.7 ± 7.35E+06	9.73E+0.5 ± 2.56E+05****	4.33E+0.7 ± 2.04E+07	2.77E+0.7 ± 7.48E+06**
T cells	2.21 ± 0.62	2.23 ± 0.38	25.55 ± 2.81	25.88 ± 2.68	1.20E+0.6 ± 5.48E+05	2.58E+0.5 ± 5.65E+04****	1.66E+0.7 ± 6.08E+06	1.70E+0.7 ± 5.08E+06
CD11b+ Gr1-	3.57 ± 1.31	5.98 ± 1.69****	1.82 ± 0.47	2.60 ± 0.67***	1.94E+0.6 ± 9.20E+05	6.97E+0.5 ± 2.28E+05****	1.19E+0.6 ± 3.95E+05	1.74E+0.6 ± 7.95E+05*
CD11b+ Gr1+	56.01 ± 7.91	69.31 ± 3.49****	2.83 ± 4.41	14.33 ± 3.33****	3.08E+0.7 ± 1.26E+07	8.22E+0.6 ± 2.11E+06****	1.64E+0.6 ± 2.16E+06	9.52E+0.6 ± 3.42E+06****

### Increased Frequencies of Hematopoietic Progenitor Cells in the *Vhl*cKO BM

**Figure 2:**
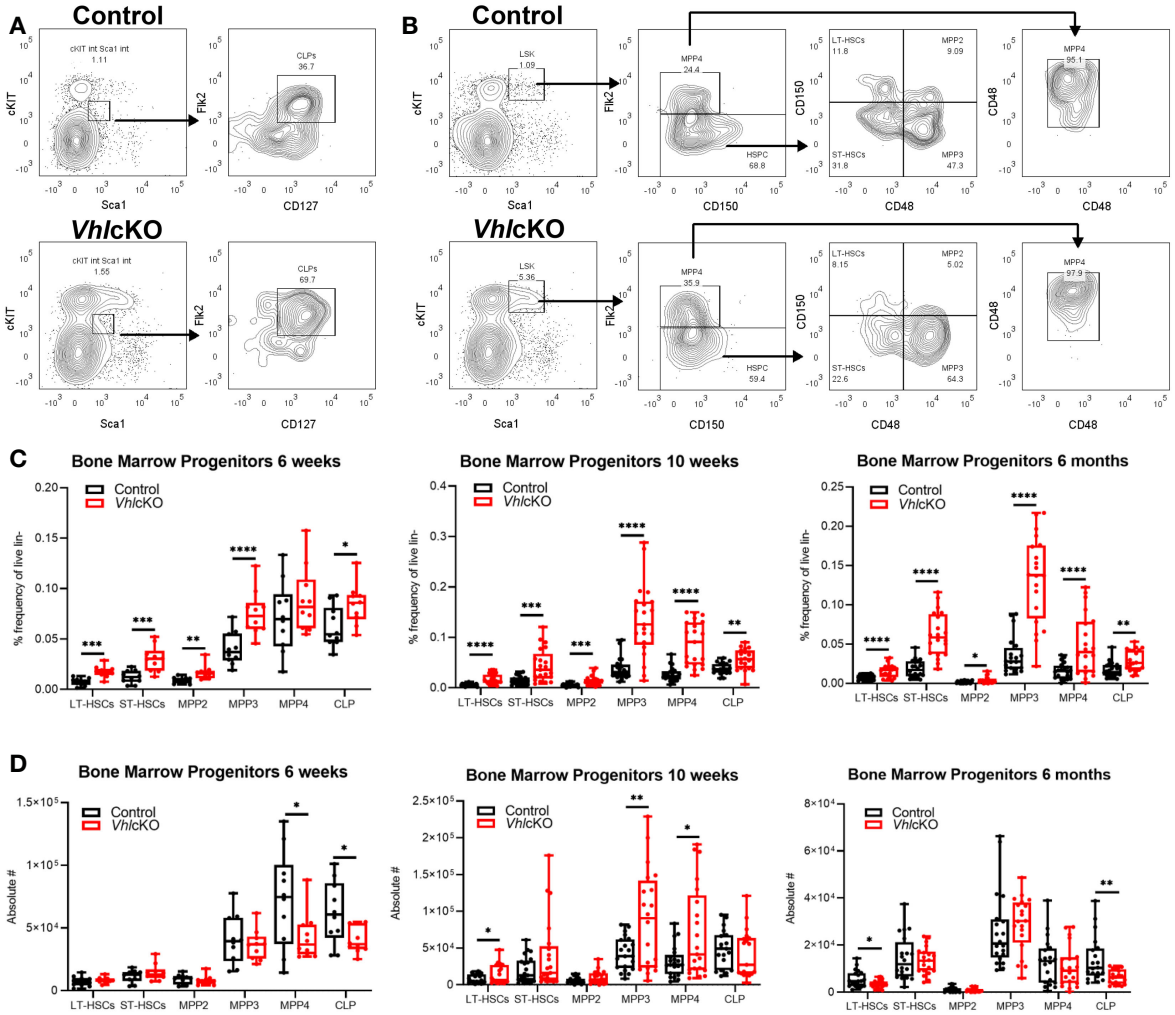
Increased frequency of hematopoietic progenitor cells in the *Vhl*cKO BM. **(A)** Representative FACS plots of common lymphoid progenitors (Lineage-, cKit^int^, Sca1^int^, CD127+ Flk2+); **(B)** representative FACS plots of hematopoietic progenitors in the bone marrow of controls (top) and *Vhl*cKOs (bottom); **(C)** frequency and **(D)** absolute number of hematopoietic progenitors in the bone marrow in 6-weeks-old (left), 10-weeks-old (middle) and 6-months-old (right) mice. p<0.05*, p<0.01**, p<0.001***, p<0.0001**** two-tailed Student’s t-test.

To further investigate if the defect in hematopoiesis occurred upstream of lineage-committed cells, we analyzed the hematopoietic progenitor compartments in the BM of *Vhl*c*KO* mice. Long-term hematopoietic stem cells (LT-HSCs: LSK, CD150+ CD48-, short term hematopoietic stem cells (ST-HSCs: LSK, CD150-, CD48-), multipotent progenitors (MPP2: LSK, CD150+, CD48+; MPP3: LSK, CD150-, CD48+; and MPP4: LSK, CD150-, Flk2+, CD48+), and common lymphoid progenitors (CLPs: Lineage-, cKit^int^, Sca1^int^, CD127+ Flk2+) from *Vhl*cKO and control mice were quantified using flow cytometry (**Figures 2A, B**). The results showed an increase in the frequency in LT-HSCs, ST-HSCs, MPP2, MPP3, and CLPs at 6-weeks, 10-weeks and 6-months-old (**Figure 2C**). MPPs are heterogeneous with different lineage-biased potential. MPP2/3 are myeloid-biased while MPP4 are lymphoid-primed (55, 56). In our results, MPP4 frequency was increased starting at 10-weeks-old (**Figure 2C**). These results show that deletion of *Vhl* in Dmp1-expressing cells increases progenitor frequencies and indicates that downstream differentiation of B cells may be blocked. However, examination of MPP4 absolute numbers showed decreased MPP4s in 6-week-old *Vhl*cKO, an increase at 10-weeks-old, and numbers similar to controls at 6-months old. In 6-week-old *Vhl*cKO mice, the absolute numbers of CLPs were decreased, in 10-week-old *Vhl*cKO mice, the absolute numbers of LT-HSCs and MPP3 were increased, whereas at 6-months-old, LT-HSCs and CLPs were decreased (**Figure 2D**).

### *Vhl* Deletion in *Dmp1*-Expressing Cells Dysregulates B Cell Development in the BM

**Figure 3:**
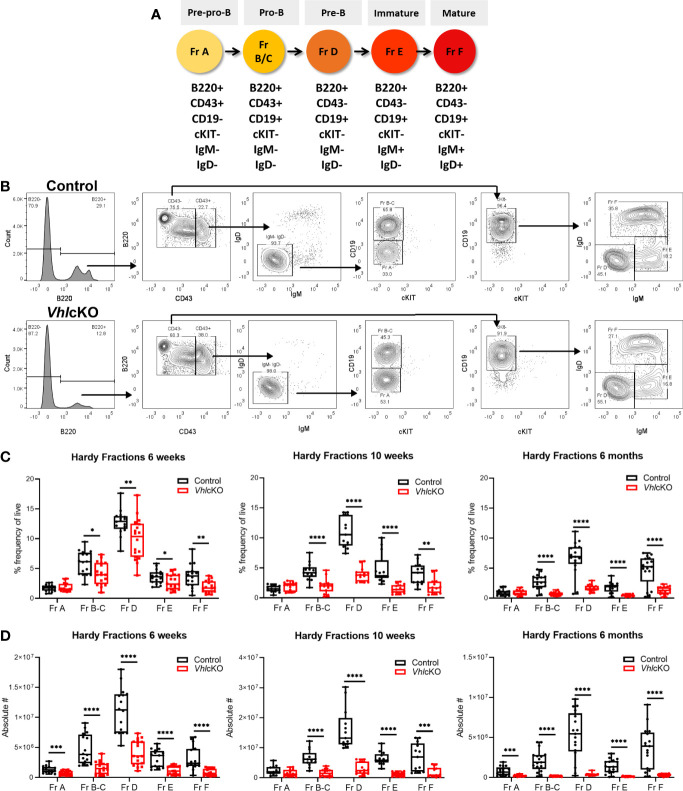
Dysregulated B cell development in *Vhl*cKO mice bone marrow. **(A)** Antigen markers used to identify and distinguish B cell development stages by flow cytometry. **(B)** Representative FACS plot of B cell development in the BM control (top) and *Vhl*cKO (bottom); **(C)** B cell frequency in 6-week-old (left), 10-week-old (middle), and 6-month-old (right) mice; **(D)** absolute cell numbers in 6-week-old (left), 10-week-old (middle), and 6-month-old (right). p<0.05*, p<0.01**, p<0.001***, p<0.0001**** two-tailed Student’s t-test.

To further explore the effects of *Vhl* deletion in OBs and OCYs on B cell development and to identify at which stage B cell development was stunted in the BM, we determined the frequencies of Hardy Fractions A-F (**Figures 3A, B**) using flow cytometry (1, 57). *Vhl*cKO mice regardless of age retained normal frequency of Fraction A. In contrast, a decrease in the frequencies of Fractions B-C through Fraction F was observed at all ages examined (**Figure 3C**). An overall decrease in the absolute numbers of B cells across all developmental stages was observed at all three ages, with the exception of Fraction A at 10 weeks (**Figure 3D**). These results indicate an incomplete but severe block in B cell development that starts at Fractions B-C in *Vhl*cKO mice.

### Reciprocal Bone Marrow Transplantation Studies Reveal a Cell-Extrinsic Effect of the *Vhl*cKO Microenvironment on B Cell Development

**Figure 4:**
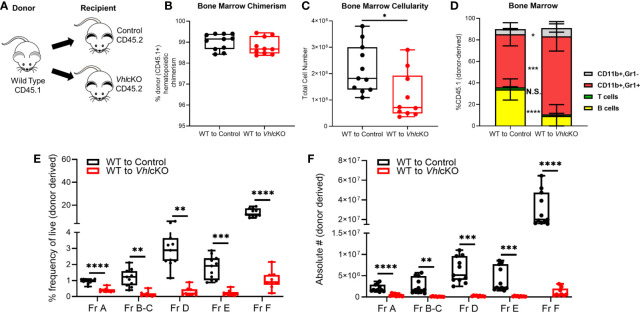
Altered B cell development in WT→*Vhl*cKO hematopoietic chimeras, demonstrating a cell-extrinsic effect of the *Vhl*-deleted osteolineage cells on B cell development. Mice were transplanted at 10 weeks of age and were analyzed 16 weeks post-transplantation. **(A)** experimental scheme; **(B)** donor (CD45.1) chimerism; **(C)** bone marrow cellularity; **(D)** frequency of lineage cells in bone marrow; **(E)** B cell frequency and **(F)** absolute number of B cell developmental stages at 16 weeks post-transplant. p<0.05*, p<0.01**, p<0.001***, p<0.0001**** two-tailed Student’s t-test. N.S., not statistically significant.

We expected the cause of the B cell defect to lie within the non-hematopoietic cells, since *Dmp1* is not expressed in hematopoietic cells. To definitively determine if the effects of *Vhl* deficiency on B lymphopoiesis were due to changes in the non-hematopoietic microenvironment within the bone, we performed whole BM transplants from WT B6.SJL (CD45.1+) donors into lethally irradiated *Vhl*cKO (CD45.2+) recipients [WT→*Vhl*cKO chimeras (**Figure 4A**)]. WT (CD45.1+)→control (Cre-negative; *Vhl^fl/fl^*, CD45.2+) chimeras were also prepared. Donor hematopoietic chimerism was similar in controls and chimeras (**Figure 4B**). Analysis 16 weeks post-transplant showed a significant reduction in BM cellularity (**Figure 4C**) and an increase in CD11b+ Gr1+ and CD11b+ Gr1- cells and a decrease in B cells in the WT→*Vhl*cKO mice (**Figure 4D**). Analysis of B cells revealed a decrease at Fractions A through Fraction F in both frequency and absolute numbers (**Figures 4E, F**), extending the defect to include Fraction A as compared to what is observed in non-transplanted *Vhl*cKO mice (**Figure 3**). In contrast, overall hematopoiesis, including B cell development, was normal in the *Vhl*cKO→WT chimeras (**Supplementary Figure 2**). Since *Vhl* deletion in B cells can affect their function (58, 59), we confirmed that *Vhl* remained intact and was not erroneously deleted in B cells in our *Vhl*cKO mice (**Supplementary Figure 3**). These results confirm a cell-extrinsic effect of the non-hematopoietic *Vhl*cKO BM microenvironment on hematopoiesis.

### *Vhl*cKO Mice Display Patterns of Reduced B Cell Proliferation and Increased B Cell Apoptosis in the BM

**Figure 5:**
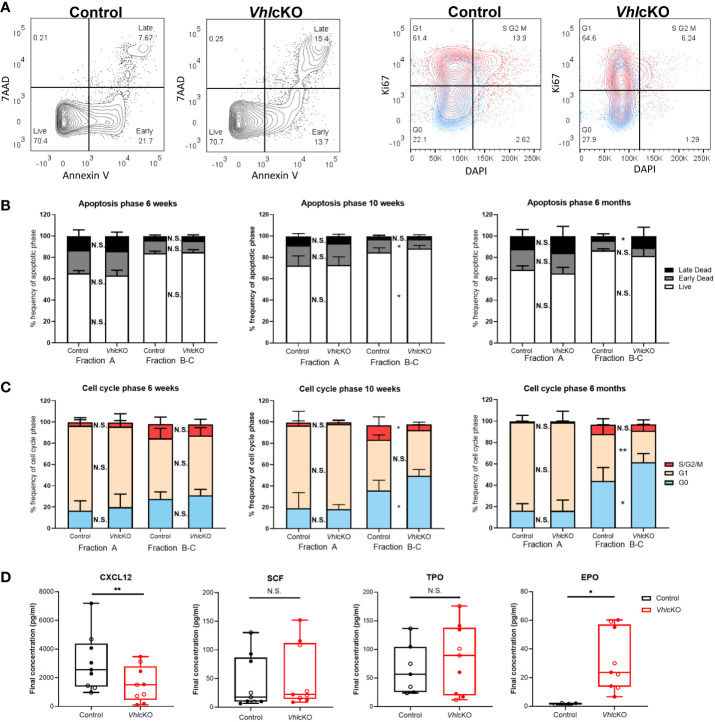
*Vhl*cKO mice display increase apoptosis and reduced cell proliferation during early B cell development. **(A)** Representative FACS plots of apoptotic phases (live, early apoptosis, and late apoptosis) in B220+ cells (left) and cell cycle phases (G0 (quiescent), G1, and S/G2/M) in B220+ cells (red:CD43+ blue: CD43-) (right) in 10-weeks-old mice; **(B)** frequency of apoptotic phases in Fractions A and B-C in 6-weeks-old, 10-weeks-old and 6-month-old mice; **(C)** frequency of cells in each cell cycle phase within Fractions A and B-C at 6-weeks-old, 10-weeks-old old and 6-month-old mice. Our cell cycle analysis antibody panel (**Supplementary Table 1**) did not include anti-IgM and anti-IgD, so we could not remove these cells from the CD43+ population. However, these cells are very low in frequency in the *Vhl*cKO (**Figure 3**) and do not significantly change the proliferation results in controls (data not shown); **(D)** CXCL12, SCF, TPO, and EPO cytokine level measurements in bone marrow supernatant of combined 10-weeks-old (filled) and 6-months-old (open) control or *Vhl*cKO mice. p<0.05*, p<0.01** two-tailed Student’s t-test. N.S., not statistically significant.

We hypothesized that the observed reduction of B cells was due to increased apoptosis and diminished B cell proliferation in the BM. To test this, B cells were stained with Annexin V and 7AAD to identify cells that were live, in early stage apoptosis or late stage apoptosis (**Figure 5A**, left panels). Normally, apoptosis is the most extensive in Fraction A (pre-pro-B cells) amongst the B cell fractions (60). The frequencies of *Vhl*cKO Fraction A cells in live, early and late apoptosis stages was comparable to controls at all ages examined (**Figure 5B**). Apoptosis in Fraction B-C in *Vhl*cKOs was similar to controls at 6-weeks-old. At 10-weeks-old, the frequency of live Fraction B-C cells increased and those in early apoptosis decreased in the *Vhl*cKO. At 6-months-old, there was no difference in the frequencies of live and early stage apoptotic Fraction B-C cells, but their frequency in late stage apoptosis was increased (**Figure 5B**). No differences in the stages of apoptosis were observed between controls and *Vhl*cKOs for Fractions D, E and F at all ages examined, with the exception of increased Fraction F cells in late stage apoptosis at 6-months-old (**Supplementary Figure 4**).

B cell development leads to the assembly and signaling of the B cell antigen receptor (BCR). CD43+ Fraction A-C (pre-pro-B and pro-B cells) normally have higher proliferation rates compared to CD43- Fraction D-E (Pre-B cells and immature B cells) (5, 61). Proliferation is halted at Fraction D (small pre-B cell) to allow light (L) chain gene rearrangement, subsequently expressing a complete IgM surface molecule (Fraction E) (5, 62). Cell cycle analysis in *Vhl*cKO B cells was performed using Ki67 and DAPI staining (**Figure 5A**, right panels). There were no differences in the distribution of cells in G0 (quiescent, DAPI- Ki67-), G1 (DAPI- Ki67+, or S/G2/M (DAPI+ Ki67+) phases between *Vhl*cKO and control mice amongst all Hardy Fractions at 6-weeks-old (**Figure 5C** and **Supplementary Figure 5**). However, at 10-weeks-old and 6-months-old, Fractions B-C contained an increased percentage of cells in G0. At 10-weeks old, a similar frequency of Fraction B-C cells in G1 was observed between *Vhl*cKO and controls, but there was a reduced percentage of cells in S/G2/M cell cycle phases (**Figures 5A, C**). At 6-months-old, this pattern reversed, with a decreased frequency of Fraction B-C cells in G1, and similar frequency of S/G2/M cells (**Figure 5C**). Taken together, these data indicate a reduced ability of Fraction B-C cells to proliferate in a *Vhl*-deficient microenvironment as early as 10-weeks-old. No difference in proliferation of Fractions D-F was observed at any age examined, with the exception of a slight (yet statistically significant) reduction of the *Vhl*cKO Fraction F cells in G0 and increase in G1 at 6-months-old (**Supplementary Figure 5**).

B cell development at each stage requires specific signaling molecules from a variety of niche cells (5, 63). To further explore the dysregulated niche, BM supernatant was analyzed for levels of CXCL12 and SCF, which are critical for B cell development (1, 27, 28, 31). CXCL12 levels were reduced in the *Vhl*cKO BM serum, while SCF levels were unaffected (**Figure 5D**). This suggested that increased apoptosis and reduced proliferation of Fraction B-C cells are caused by reduced CXCL12 levels in the *Vhl*cKO BM.

### Increased Bone Marrow Blood Vessel Diameter and Density in *Vhl*cKO Microenvironments

**Figure 6:**
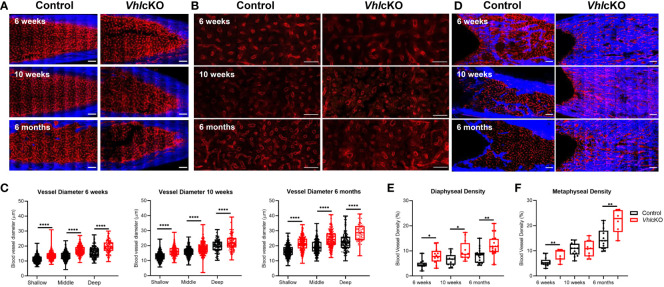
| *Ex vivo* two-photon imaging of long bones in *Vhl*cKO and controls. **(A)** Representative macroscopic images of the femur diaphyseal BM (scale bars: ~200 μm), **(B)** magnified z-stacks (scale bars: ~100 μm), and **(C)** statistical analysis after uDISCO clearing show an increase in the *Vhl*cKO vascular diameter relative to the controls; **(D)** *ex vivo* images of femur metaphyseal BM after max intensity projection reveal bone replacement and vascular alteration in *Vhl*cKO; **(E, F)** quantification of the metaphyseal and diaphyseal vascular density (scale bars: ~200 μm). Red: blood vessels (labeled with Alexa647 conjugated antibodies against CD31, CD144, and Sca-1), Blue: bone (SHG: Second harmonic generation). *p<0.05, **p<0.01, ****p<0.0001, two-tailed Student’s t-test.

We attempted to quantify MSC, OB and EC subsets using flow cytometry of collagenase-digested bones (64), but we concluded that the high bone mass of *Vhl*cKO mice prevented complete digestion to accurately enumerate these populations (**Supplementary Figure 6**). To more precisely examine the changes in the microenvironment of *Vhl*cKO mice, we imaged femurs that were shaved to remove cortical bone (for analysis of the metaphysis) or optically cleared with a modified uDISCO protocol (for analysis of the fully intact diaphysis) **(Supplementary Videos 1, 2)** (49). We measured the vessel diameter and frequency in the cleared long bones and found that regardless of their position in the BM, blood vessels in *Vhl*cKO mice were significantly larger in diameter than the control group (**Figures 6A**–**C**) while generally no difference was observed in the vessel frequency (**Supplementary Figure 7A**). Metaphyseal and diaphyseal BM and bone vessel density measurements revealed that in *Vhl*cKO, blood vessels occupy a larger volume than controls (**Figures 6D**–**F** and **Supplementary Figure 7B, C**). Furthermore, we observed an apparent decrease in endosteal lining arterioles in the diaphysis of 6-month-old *Vhlc*KO femurs compared to controls (**Supplementary Figure 7D**). Taken together, these data reveal a striking alteration in the overall architecture of the BM vascular network in *Vhlc*KO mice.

**Figure 7:**
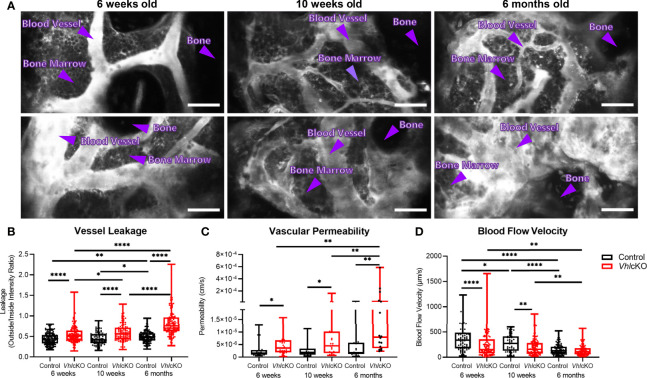
Disruption in blood-bone marrow barrier revealed by intravital microscopy. Blood vessel microenvironment comparisons of control and *Vhl*cKO mice at 6-week (n=4), 10-week (n=4) and 6-month (n=5) timepoints. **(A)** Representative contrast adjusted max intensity projections of the calvarial BM in control and *Vhl*cKO mice by age; White: blood vessel (Rhodamine B Dextran, 70 kDa); scale bar: 50 μm; quantification of calvarial BM **(B)** blood vessel leakage, **(C)** vascular permeability, and **(D)** blood flow velocity. *p<0.05, **p<0.01, ****p<0.0001, Mann-Whitney test.

### *Vhl*cKO Bone Marrow Blood Vessels Display Increased Permeability

While it has been shown that the bone and vascular system undergoes significant remodeling in *Vhl*cKO mice, there has been a lack of information regarding potential functional changes to BM blood vessels. To examine changes to the BM vasculature system which could negatively impact B cell development, we sought to quantify changes to the vascular permeability, leakage and blood flow velocity *via* intravital two-photon microscopy of the calvaria. Vessel permeability reflects the rate at which small molecules exit blood vessels and fill the surrounding perivascular space, whereas leakage is the ratio of fluorescent dye in the perivascular space and vascular lumen after reaching equilibrium. Blood vessel leakage and permeability was calculated by administering Rhodamine B Dextran (70kDa) *via* a retro-orbital injection. We found that *Vhl*cKO mice displayed greater vascular leakage overall, and that vascular leakage increased in both control and *Vhl*cKO mice with age (**Figures 7A, B** and **Supplementary Videos 3**–**8**). Similarly, we observed an increase in vascular permeability in *Vhl*cKO mice, which significantly increased with age (**Figure 7C** and **Supplementary Videos 9, 10**). We observed a decrease in blood flow velocity in *Vhl*cKO mice compared to controls for 6-week-old and 10-week-old mice (**Figure 7D**). Lastly, we observed an age-related reduction in blood flow in both *Vhl*cKO and control mice (**Figure 7D**), which is consistent with previously published changes in BM vascular flow rate with age (65).

### Evidence for Age-Related Reduction in Oxygen Levels Within Local Niches in the *Vhl*cKO Bone Marrow

**Figure 8:**
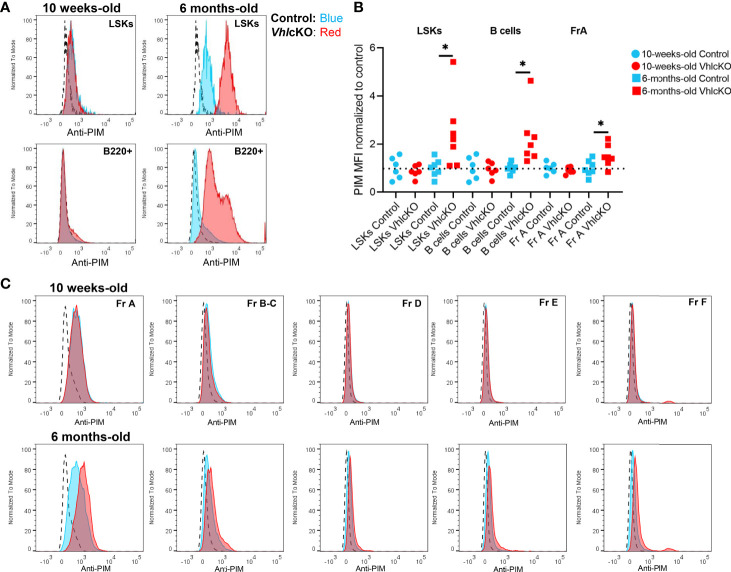
Hypoxia cell marker pimonidazole indicates difference by age and amongst B cell fractions in control and VhlcKO mice. *Vhl*cKO and control mice were injected with PBS or 120 mg/kg pimonidazole (PIM). PIM staining of **(A)** Live, Lin-, CD45+, Sca1+, cKit+ (LSKs) (top) and Live, B220+ cells (bottom) in the BM of 10-weeks-old and 6-months-old mice; dashed line: isotype control; blue line: anti-PIM staining in controls; red: anti-PIM staining in *Vhl*cKO; **(B)** summary of PIM staining in *Vhl*cKOs (red) normalized to the mean fluorescence intensity (MFI) in controls (blue); results from 4 independent experiments are shown; **(C)** representative anti-PIM staining plots of individual B cell Fractions **(A–F)** from a control (blue) and a *Vhl*cKO mouse (red) at 10-weeks-old (top) and at 6-months-old (bottom). *p<0.05, two-tailed student's t-test.

Hypoxic niches in the BM microenvironment are crucial for hematopoietic development but BM oxygenation can be altered through changes in vascular supply and/or cellular consumption (11). Dynamic regulation of HIF-1α levels is required for normal B cell development such that HIF activity is high in B cell precursors and must decrease in the immature B cell stage in the BM (66). In wild type mice at 10-16 weeks of age, studies using the hypoxic marker pimonidazole (PIM) revealed that HSCs in the BM stain positively with PIM, indicating a hypoxic niche (67). In contrast, low PIM staining in BM B220+ cells was observed in 6-12 week old mice, indicating a relatively normoxic niche for B220+ cells in wild type mice (68). To evaluate hypoxia in distinct B cell developmental stages, *Vhl*cKO and control mice were injected with PBS or 120 mg/kg PIM. PIM staining of LSKs in the BM was positive, as previously reported (67), but this staining was more intense in LSKs of control and *Vhl*cKO mice at 6 months of age (**Figure 8A** top panels). Remarkably, PIM staining in *Vhl*cKO LSKs was significantly higher than control LSKs at 6 months (**Figure 8B**). CD45+ B220+ cells [which include all Hardy Fractions, in addition to other hematopoietic progenitors, natural killer cells, dendritic cells and T cells (69–73)] displayed negative or low staining with PIM in both control or *Vhl*cKO mice at 10 weeks old, but the PIM staining in B220+ cells in *Vhl*cKO mice at 6 months was significantly elevated compared to controls (**Figure 8A**, bottom panels and **Figure 8B**). Next, we performed PIM staining in order to determine if specific Hardy Fractions were experiencing hypoxia in the *Vhl*cKO bone marrow. This analysis revealed that in general, the Fraction A cells stain with PIM at a higher level than the Fractions B through Fraction F cells (**Figure 8C**), but that the intensity of PIM staining on Fraction A cells in the *Vhl*cKO mice at 6 months of age was significantly higher than controls (**Figures 8B, C** and **Table 2**). This reveals that in wild-type mice, Fraction A cells might reside in a hypoxic niche, similar to LSKs. It also indicates that as B cells mature, they may move away to a less hypoxic niche. Our results also indicate that the oxygen levels in the microenvironment of Fraction A cells in the *Vhl*cKO BM is similar to controls at 10-weeks-old, but at 6-months-old, the microenvironment for Fraction A cells is relatively hypoxic compared to the microenvironments for Fraction B through Fraction F.

**Table 2:** Mode Fluorescence Intensity of PIM staining on B cell fractions in control and *Vhl*cKO mice.

Age	Genotype	Treatment	MFI (mode)				
			Fr A	Fr B-C	Fr D	Fr E	Fr F
**10-weeks-old**	*Vhl* cKO	Isotype	336	187	146	125	146
	*Vhl* cKO	Isotype	358	166	146	166	208
	Control	PIM	3561	1177	638	638	613
	Control	PIM	3678	1501	689	638	689
	Control	PIM	2940	742	470	493	402
	Control	PIM	3926	1547	715	824	663
	Control	PIM	1371	293	229	229	229
	Control	PIM	715	250	187	187	187
	*Vhl* cKO	PIM	2514	796	380	402	424
	*Vhl* cKO	PIM	3034	912	493	540	588
	*Vhl* cKO	PIM	3132	1371	493	564	564
	*Vhl* cKO	PIM	3561	943	540	564	516
	*Vhl* cKO	PIM	1106	424	336	336	358
	*Vhl* cKO	PIM	882	271	208	187	208
**6-months-old**	Control	PIM	293	146	104	125	125
	*Vhl* cKO	PIM	293	83.5	83.5	125	146
	Control	PIM	4057	1290	613	689	613
	Control	PIM	1413	470	358	380	358
	Control	PIM	1744	516	336	358	336
	Control	PIM	2292	447	336	336	358
	Control	PIM	1413	493	314	358	314
	Control	PIM	882	293	208	229	229
	Control	PIM	1006	336	229	250	229
	*Vhl* cKO	PIM	3800	1106	824	742	882
	*Vhl* cKO	PIM	2292	742	424	564	613
	*Vhl* cKO	PIM	4481	1594	769	1038	974
	*Vhl* cKO	PIM	3926	1106	824	796	974
	*Vhl* cKO	PIM	1594	493	271	271	336
	*Vhl* cKO	PIM	1594	516	293	336	424
	*Vhl* cKO	PIM	1330	271	293	271	380

## Discussion

Here, we report that deletion of the *Vhl* gene in *Dmp1*-expressing cells results in cell-extrinsic changes in the bone marrow microenvironment that deleteriously affect B cell development as early as 6 weeks of age. Specifically, we observed reduced CXCL12 levels in the bone marrow, which could result in the inability of Fraction B-C to proliferate. We also observed elevated levels of EPO, and an increase in the blood vessel diameters and vessel density in the *Vhl*cKO at all ages examined, consistent with a response to hypoxia. To our knowledge, our report is the first to show pimonidazole binding on Fraction A cells in wild type mice, indicating that in general, Fraction A cells reside in hypoxic niches of the BM, similar to LSKs. Burrows et al., 2020 utilized EF5, a hypoxia probe similar to pimonidazole (66), and reported high EF5 staining of “pro-B/pre-B” (B220+ IgM- IgD-) cells, which includes Hardy Fractions A-D, but they did not distinguish EF5 staining on clearly delineated Hardy Fractions, as we have in our current study. In addition, our staining of Hardy Fraction cell subsets with pimonidazole revealed that Fraction A cells in the *Vhl*cKO experienced more extreme hypoxia at 6 months of age. Collectively, our analyses demonstrate that the B cell developmental defects in the *Vhl*cKO bone marrow microenvironment observed at younger ages (6 weeks and 10 weeks) are not due to dysregulation of oxygen levels in their local niches. However, the B cell defects could be exacerbated by hypoxia as the mice age to 6 months.

Evidence from several groups, including our own (2, 47, 74) supports that distinct BM cell subsets, including perisinusoidal cells (which are a subset of MSCs), osteoprogenitor cells (OBPs), OBs and OCYs support different stages of B cell maturation by providing CXCL12 (75, 76) and IL7 (1), both of which are important for proliferation and survival of Hardy Fractions A, B and C (a.k.a. pre-pro-B and pro-B cells) (4). Hematopoietic stem cells and progenitors are localized in the relatively hypoxic sinusoidal regions of the marrow (11, 12) which are anatomically and physically separate from the endosteal niches. Osteolineage cells originate from MSCs, which then differentiate to OBPs, early OBs, late OBs and mature OCYs. MSCs and HSCs are found in close proximity to each other (77) and might also be located within the BM cavity in direct contact with B cell progenitors (1). Osteoblast depletion studies *in vivo* demonstrated OBs as a key regulator of B cell development (78) and this was later supported later by independent studies in mice, in which OBs that lack expression of Gsα (79) and that OBs defective in the mTORC1 signaling pathway (80) could not support full B cell development. The role of MSCs in the regulation of B cell proliferation, survival, and differentiation appears to be highly context-dependent (81–83), and new reports of novel CD51+ MSC subsets and their differential ability to support B lymphopoiesis in the BM (25, 37) will require further scrutiny in the context of altered bone homeostasis.

One caveat to the identification of the “true” niche cells that support B cell development is new information on off-target gene deletion in *Dmp1*-Cre mice. We utilized *Dmp1*-Cre for our *Vhl* deletion studies as they are the main model currently available to target osteocytes. However, despite its widespread use, *Dmp1-Cre* displays off-target expression (43, 84, 85). Broad MSC targeting of *Vhl* in *Prx-Cre;Vhl^fl/fl^* mice resulted in delays in BM cavity development, increases in trabecular bone with dilated BM vessels and few hematopoietic cells in perinatal mice (86). Similar phenotypes were observed in *Osx-Cre;Vhl^fl/fl^* mice (87), perhaps because *Osx* and *Prx* expression overlap at an early osteoprogenitor stage. *Ocn-Cre;Vhl^fl/fl^* mice, which targets mainly mature OBs, displayed similar bone and hematopoietic phenotypes plus angiogenesis in the long bones and changes in OCY morphology with fewer dendrite connections (23). Taken together, these studies indicate that deletion of *Vhl* at the MSC, OBs and OCY phases from ontogeny results in physical changes in bone microenvironment and altered hematopoiesis, and implies that the phenotypes observed could have been generated at an early osteoprogenitor stage and erroneously attributed to more mature osteolineages. Single cell RNA-Seq data on bone marrow stromal cells (88–90) could provide information on non-overlapping mRNAs between MSCs, early OBs, late OBs, in order to create new mouse models for studies of HSC and B cell bone marrow niches, and permit discovery of the specific contributions of MSCs and OBPs to B cell development.

Our studies show an effect of *Vhl*-deletion in *Dmp1*-expressing cells on ECs. Our imaging results suggest that there is an increase in bone ECs, which is consistent with previous studies in *Osx-*Cre;*Vhl^fl/fl^* mice where endomucin staining showed that *Vhl* deletion increased bone vasculature with dilated blood vessels (21). We also observed larger vessels in the BM across all ages and an increase in BM blood volume. These changes, along with the observed decrease in endosteal arterioles in the long bones of 6 month old mice and an increase in PIM staining, suggests that oxygenation of the *Vhl*cKO marrow may be lower than normal, which may play a role in dysregulation of B cell development in older mice. Future studies will be needed to clarify this and to identify other changes in specific types or locations of blood vessels in the *Vhl*cKO model as a function of age.

Given the connection between *Vhl* and hypoxia response, it was interesting that EPO levels were high in the BM supernatant of the *Vhl*cKO mice. High *Epo* mRNA was also observed in the bones of *Osx*-Cre;*Vhl^fl/fl^* (8) mice. Deletion of *Vhl* at the mature OB stage using the *Osx*-Cre (8) and *Ocn-*Cre (22) (targeting osteoprogenitors) and in MSCs, OBs and OCYs using *Dmp1*-Cre (23), increased bone mass and angiogenesis, likely through HIF1α-regulated expression of VEGF and EPO. If elevated EPO levels directly affect B cell development in the *Vhl*cKO BM has not yet been verified. However, it has been reported that ECs in the BM suppress levels of CXCL12 expression in response to increased EPO levels (91). We also observed decreased CXCL12 in the BM supernatant of *Vhl*cKO mice. CXCL12 is required for proper development and retention of B cells in the BM (29, 76). This suggests that altered vascular components in the *Vhl*cKO bone and BM microenvironments impair B cell development possibly through the effects of EPO on EC function.

Permeability of the BM vasculature in the *Vhl*cKO mice was also compromised. We found an increased vascular leakage and permeability in the *Vhl*cKO BM compared to controls regardless of age. In addition, vascular permeability appeared to increase with age, with the highest vascular permeability and leakage being observed in 6-month-old *Vhl*cKO mice when compared with 6-week-old mice. Interestingly, it was observed that vascular blood flow velocity decreased in 6-week-old and 10-week-old *Vhl*cKO mice but was not affected in 6-month-old *Vhl*cKO mice. An increase in blood flow velocity would normally explain an increase in permeability and leakage, but that is not evident in our data. Instead, the more likely explanation is that the blood-bone marrow barrier is compromised, increasing the exposure of the BM to plasma components.

Deletion of *Vhl* in B cells stabilizes *Hif1α* levels and affects mature B cell function by impairing cell proliferation, antibody class-switching, generation of high affinity antibodies, antibody responses, and impairs metabolic balance essential for naive B cell survival and development (58, 59, 92). Dynamic regulation of HIF-1α levels was also found to be a crucial step in B cell development in the BM (66). Burrows et al. found decreased *Hif*1α activity at the immature B cell stage in the BM and that HIF-1α suppression was required for normal B cell development (66). This dynamic regulation of HIF-1α activity during B cell development is consistent with our results, which revealed that Fraction A cells stain highly with PIM, and PIM staining was reduced as B cell development progressed to Fraction F. Together, our findings and that of Burrows et al. suggest that the earliest B cell stages (e.g. pre-pro B, Fraction A) might prefer a more hypoxic niche compared to the later B cell stages. Although *Vhl* is deleted in *Dmp1*-expressing cells in our model, we cannot yet rule out if this deletion is artificially causing changes that would be found in a true hypoxic state through *Hif1* stabilization, when in fact the oxygenation of the BM of the *Vhl*cKO is not altered. In addition, PIM cannot provide true quantification of dissolved oxygen concentration in tissue. PIM adduct staining results could reflect inadequate oxygen supply to the BM, faulty rates of intracellular oxygen consumption, or both. Direct *in vivo* measurement of oxygen tension using two-photon phosphorescence lifetime microscopy would help answer this question (11).

**Figure 9:**
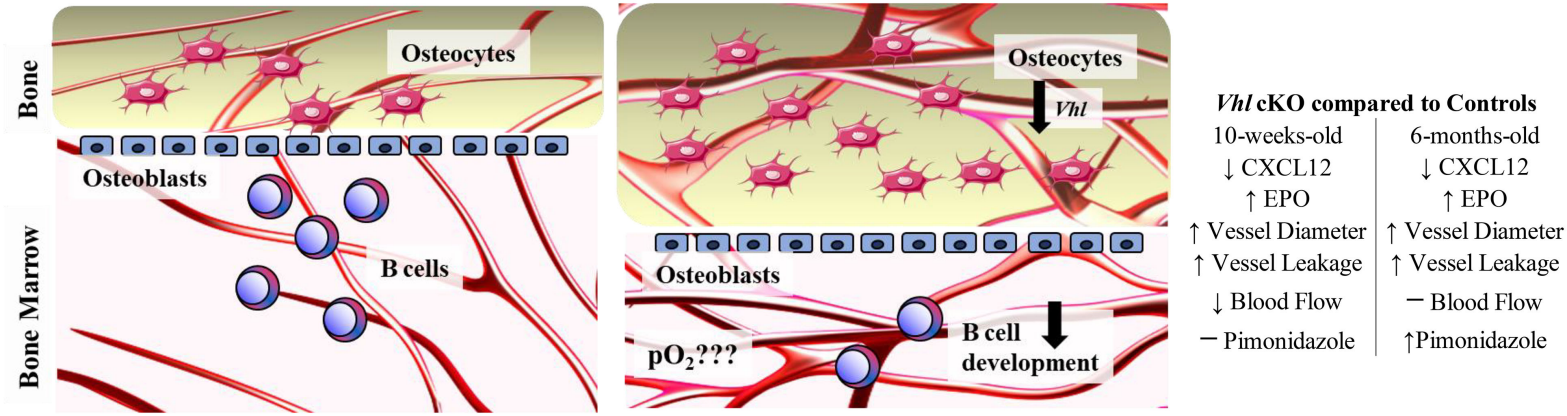
Working model describing the changes in the bone marrow microenvironment in *Vhlc*KO mice. Left panel: Schematic of healthy control bone marrow where VHL/HIF signaling is intact, the transition from osteoblasts to osteocytes is homeostatically balanced and interactions of developing B cells and stromal cells within their niches promotes their differentiation, maturation and proliferation. Right panel: Lack of *Vhl* in late osteoblasts and osteocytes has a severe effect on hematopoiesis in the bone marrow, changing the B cell niche and indirectly regulating B cell development through decrease of CXCL12, increase of EPO and changes to the BM microenvironment vasculature and permeability. Changes in the oxygen levels in the local niches for Fraction A cells do not occur until later ages, as shown by pimonidazole staining. Direct measurement of pO_2_ in the BM is necessary to determine if the BM oxygenation landscape is altered compared to controls.

The information generated in this study helps define the role of *Vhl* and altered bone homeostasis on immune cell development. Our results suggest the following working model of the interactions in the BM microenvironment that controls B cell development (**Figure 9**): *Vhl* in *Dmp1*-expressing MSCs, OBs and OCYs plays a significant role in the BM microenvironment, indirectly regulating B cell development through a decrease in CXCL12, an increase in EPO, increased vasculature and vascular permeability. However, the oxygen levels in the *Vhl*cKO appear to be dynamic, such that developing Fraction A cells experience hypoxia in older, but not younger mice. We conclude that the B cell developmental defects in the BM of *Vhl*cKO mice are not initiated by dysregulated oxygen levels in the BM. However, direct measures of oxygen tension in the local niches of each Hardy B cell Fraction is yet to be performed (and is a goal for our future studies). Our results demonstrate the significant changes of the physical niche in *Vhl*cKO mice and their effects on B cell development. Whether the physical space, niche cells, or molecular signals all play a direct or indirect role on B cell development remains to be explored and defined, with the possibility that these events are completely independent of each other. The results of this work could contribute to the development of new therapies or new targets for exogenous CXCL12 and EPO antagonists, to preserve and improve bone marrow function during microenvironmental niche changes or stress.

## Data Availability Statement

The raw data supporting the conclusions of this article will be made available by the authors, without undue reservation.

## Ethics Statement

The animal study was reviewed and approved by University of California, Merced IACUC.

## Author Contributions

BC, NA, CB, JS, and JM contributed to experimental design, data collection, analysis, and manuscript writing. HT contributed to data collection and analysis. JS and JM approved the final manuscript and are joint senior authors.

## Funding

This work was funded by the University of California, NIH grants R15 AI154245-01 (JM and JS) and NIH F31 AI154815 (BC), and through support of the NSF-CREST: Center for Cellular and Biomolecular Machines at the University of California, Merced (NSF-HRD-1547848; NA, CB, and JS).

## Conflict of Interest

The authors declare that the research was conducted in the absence of any commercial or financial relationships that could be construed as a potential conflict of interest.

## Publisher’s Note

All claims expressed in this article are solely those of the authors and do not necessarily represent those of their affiliated organizations, or those of the publisher, the editors and the reviewers. Any product that may be evaluated in this article, or claim that may be made by its manufacturer, is not guaranteed or endorsed by the publisher.

## Supplementary Material

Supplementary Videos 1, 2 Representative ex vivo Videos recorded in the uDISCO cleared long bone BM.

Supplementary Video 1 Representative 10-week-old Control uDISCO cleared long bone Z stack (scale bar ~200 μm).

Supplementary Video 2 Representative 10-week-old Control uDISCO cleared long bone 3D view (scale bar ~100 μm).

Supplementary Videos 3-8 Representative Leakage Videos recorded in the calvaria BM. Representative zstacks of the calvaria BM recorded 10 minutes after Rhodamine B Dextran injection. Z step size is 2 μm and scale bars ~50 μm. Green Channel = bone (SHG), Blue = Rhodamine-B-Dextran (70 kDa). Brightness/Contrast adjusted for display only.

Supplementary Video 3 Representative 6-week-old Control Leakage Zstack.

Supplementary Video 4 Representative 6-week-old VhlcKO Leakage Zstack.

Supplementary Video 5 Representative 10-week-old Control Leakage Zstack.

Supplementary Video 6 Representative 10-week-old VhlcKO Leakage Zstack.

Supplementary Video 7 Representative 6-month-old Control Leakage Zstack.

Supplementary Video 8 Representative 6-month-old VhlcKO Leakage Zstack.

Supplementary Videos 9, 10 Representative permeability videos recorded in the calvaria BM. Representative video of the calvaria BM permeability recorded immediately after Rhodamine-B-Dextran injection. Scale bars ~50 μm. Brightness/Contrast adjusted for display only.

Supplementary Video 9 Representative 6-week-old Control Permeability Video.

Supplementary Video 10 Representative 6-week-old VhlcKO Permeability Video.
