# Supplementary material for: Deletion of Vhl in Dmp1-Expressing Cells Causes Microenvironmental Impairment of B Cell Lymphopoiesis
Source: Front Immunol. 2022 Feb 16;13:780945. doi: 10.3389/fimmu.2022.780945 (PMC8889104; doi:10.3389/fimmu.2022.780945)
Supplement: Supplementary file 1 [file DataSheet_1.pdf]

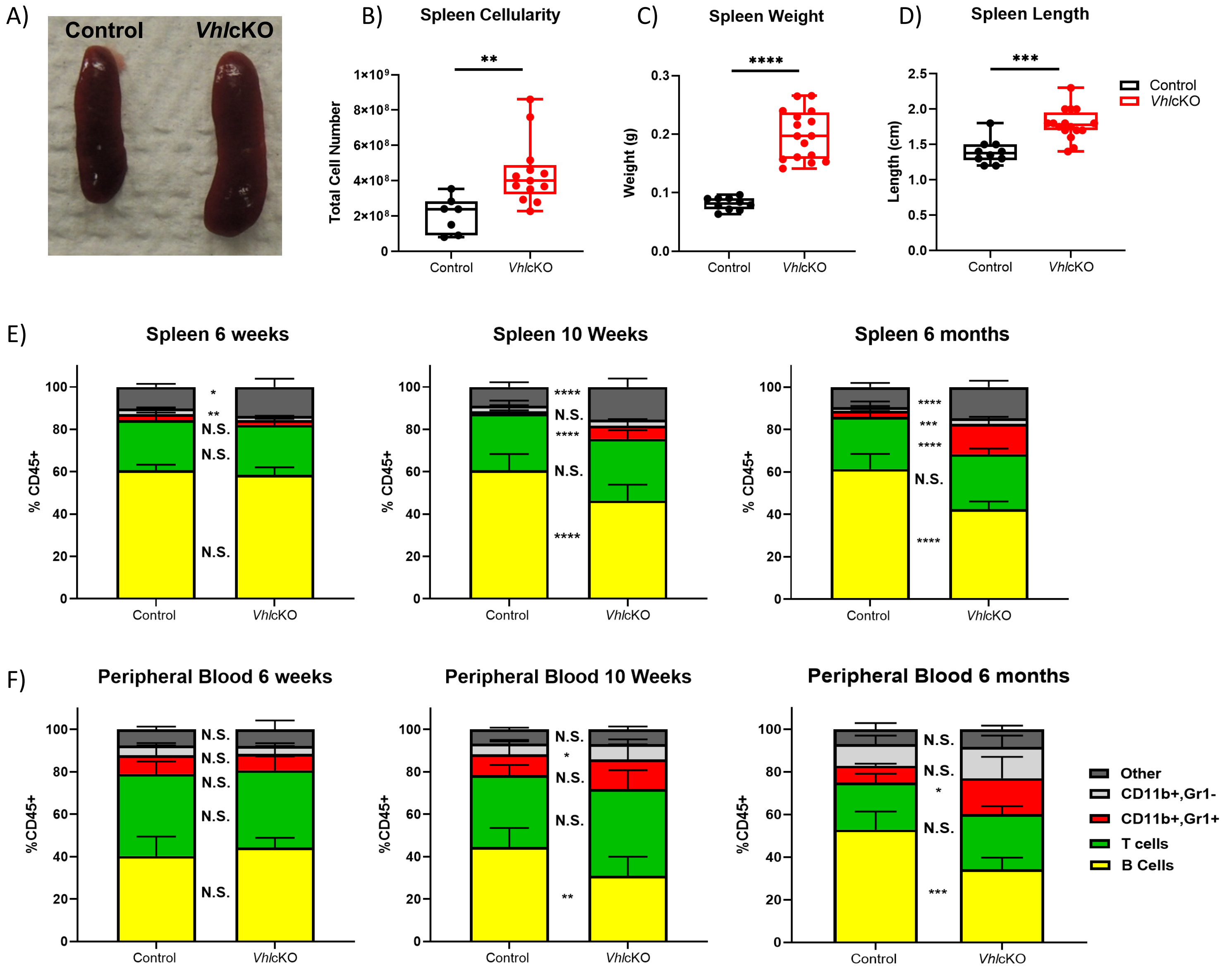

Supplemental Figure 1. Analysis of hematopoietic lineages and cellularity in the spleen of *Vhlc*KO mice. A) White light image of spleens, scale bar: 1cm; B) spleen cellularity; C) spleen weights; D) spleen length; E) spleen and F) peripheral blood lineage frequency at 6-weeks-old (left), 10-weeks-old (middle), and 6-months-old (right). *p<0.05, **p<0.01,***p<0.001, ****p<0.0001, two-tailed Student's t-test.

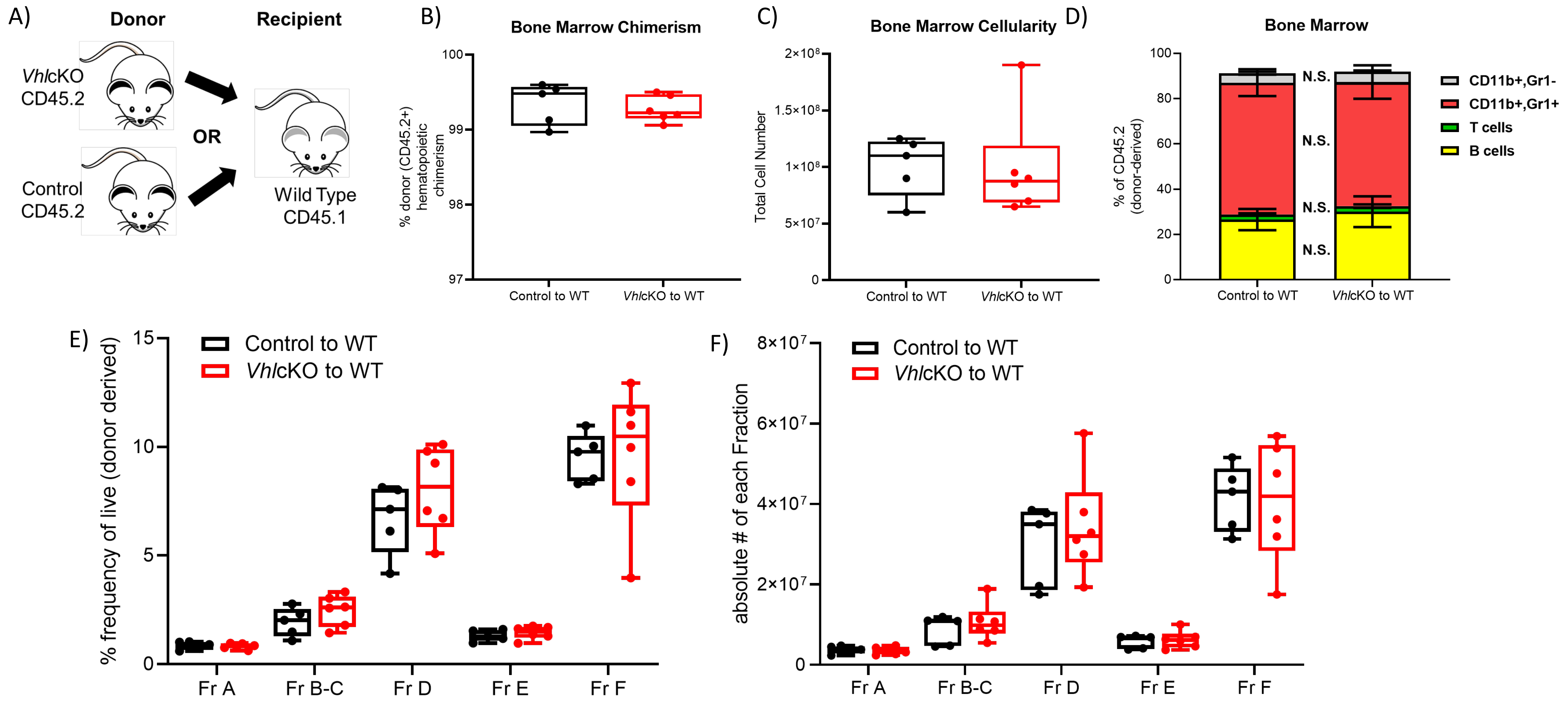

Supplemental Figure 2. Evidence against cell-intrinsic effects of *Vhl* deletion on B cell development in *Vhl*cKO mice. A) Experimental scheme. Mice were transplanted at 10 weeks of age and were 26 weeks old at time of analysis. B) donor (CD45.2+) chimerism; C) bone marrow cellularity; D) frequency of lineage cells in bone marrow; E) frequency and F) absolute numbers of B cell developmental stages in chimeras 16 weeks post-transplant. N.S. not statistically significant; two-tailed Student's t-test.

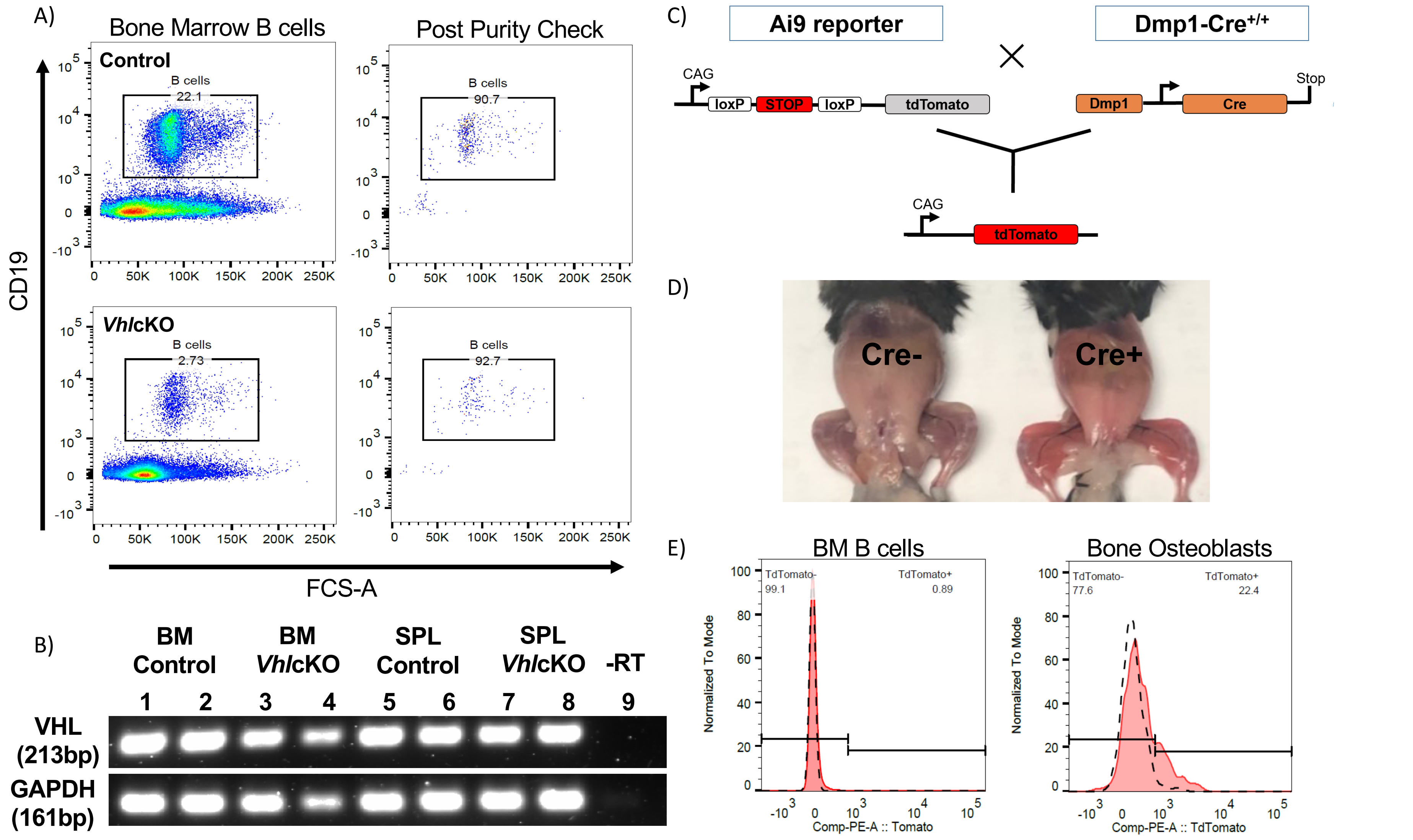

Supplemental Figure 3. Validation of *Vhl* expression in B cells in *Vhl*cKO mice and expression of *Dmp1*-Cre using Ai9 reporter mice A) Bone marrow CD19+ B lymphocyte percentages pre- and post- sorting from control and *Vhl*cKO mice and B) PCR validation of *Vhl* and *Gapdh* gene expression in DNA of sorted B cells (Live, CD19+), demonstrating *Vhl* is intact in B cells in the *Vhl*cKO; C) reporter cross of *Dmp1*-Cre mice with Ai9 (tdTomato) mice; D) tdTomato expression in *Dmp1*-Cre;Ai9 mice; E) flow cytometry measurement of tdTomato on BM B cells and osteoblasts (Lin-, CD45-, CD31-, Sca1-, CD51+) in *Dmp1*-Cre;Ai9 mice.

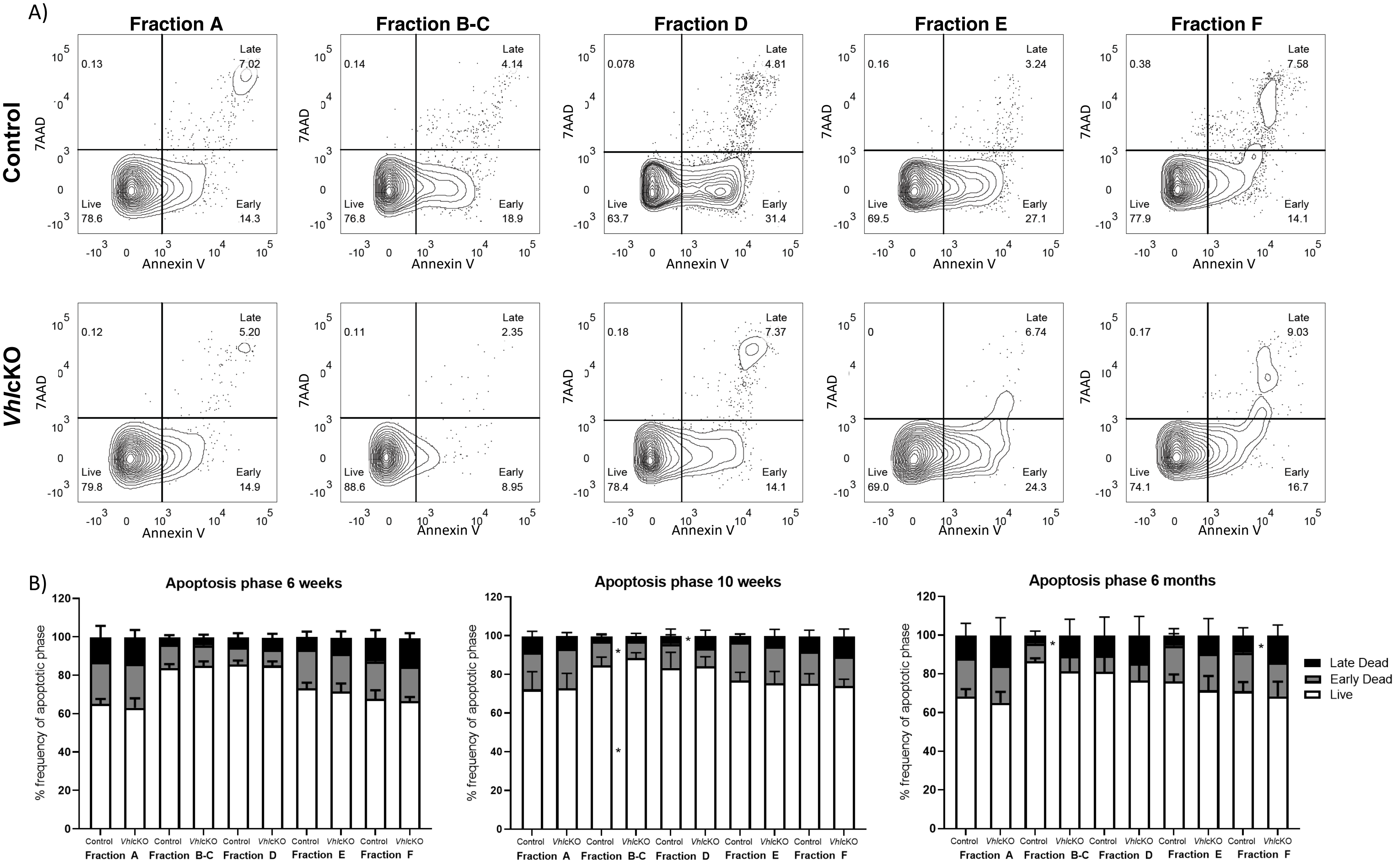

Supplemental Figure 4. Apoptosis analysis of B cell Fractions A-F A) Representative FACS plot of apoptotic phases in control (top) and *Vhl*cKO (bottom) at 10-weeks-old; B) frequency of apoptotic phase in Fractions A-F in 6-weeks-old, 10-weeks-old and 6-month-old mice; $p<0.05$*; two-tailed Student's t-test.

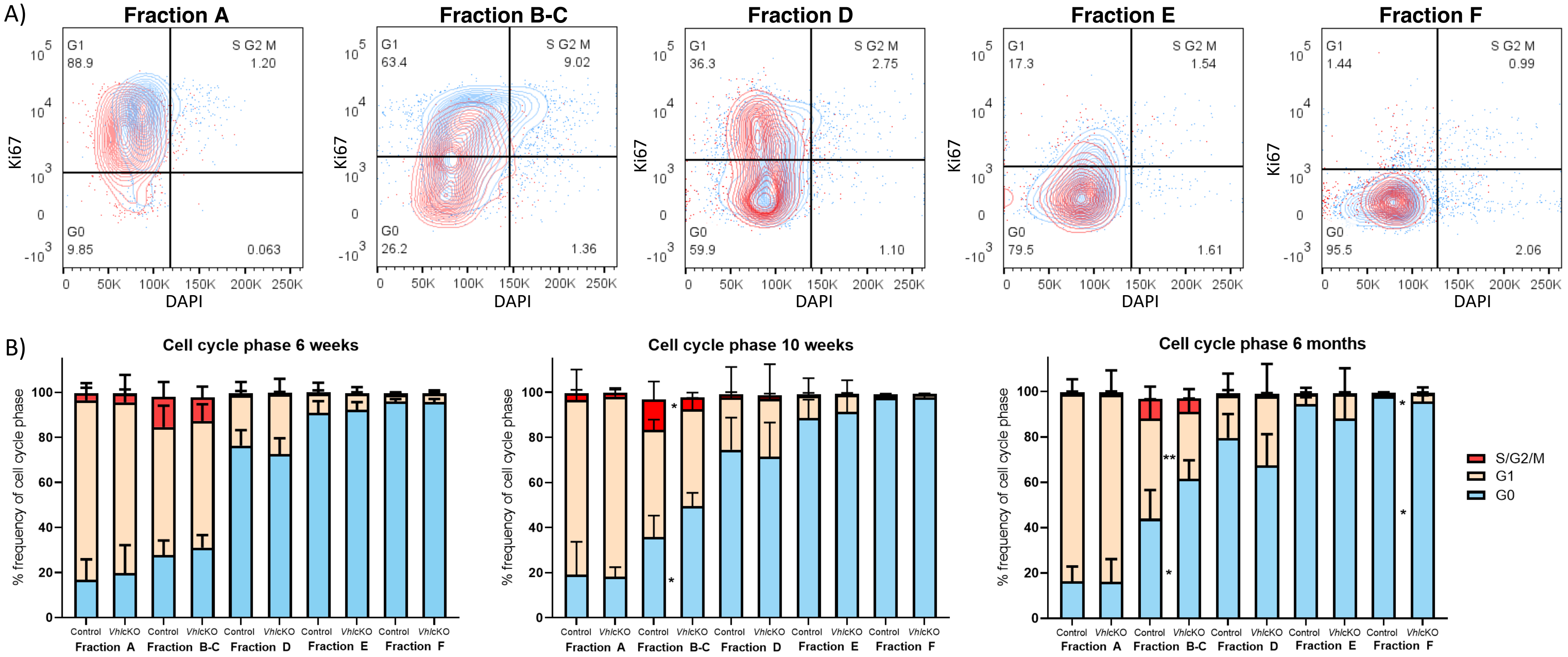

Supplemental Figure 5. Proliferation analysis of B cell Fractions A-F A) Representative FACS plot of cell cycle analysis in Fractions A-F cells (red: *Vhl*cKO blue: control) in 10-weeks-old mice; B) frequency of cells in each cell cycle phase within Fractions A-F at 6-weeks-old, 10-weeks-old old and 6-month-old mice. p<0.05*, p<0.01**; two-tailed Student's t-test.

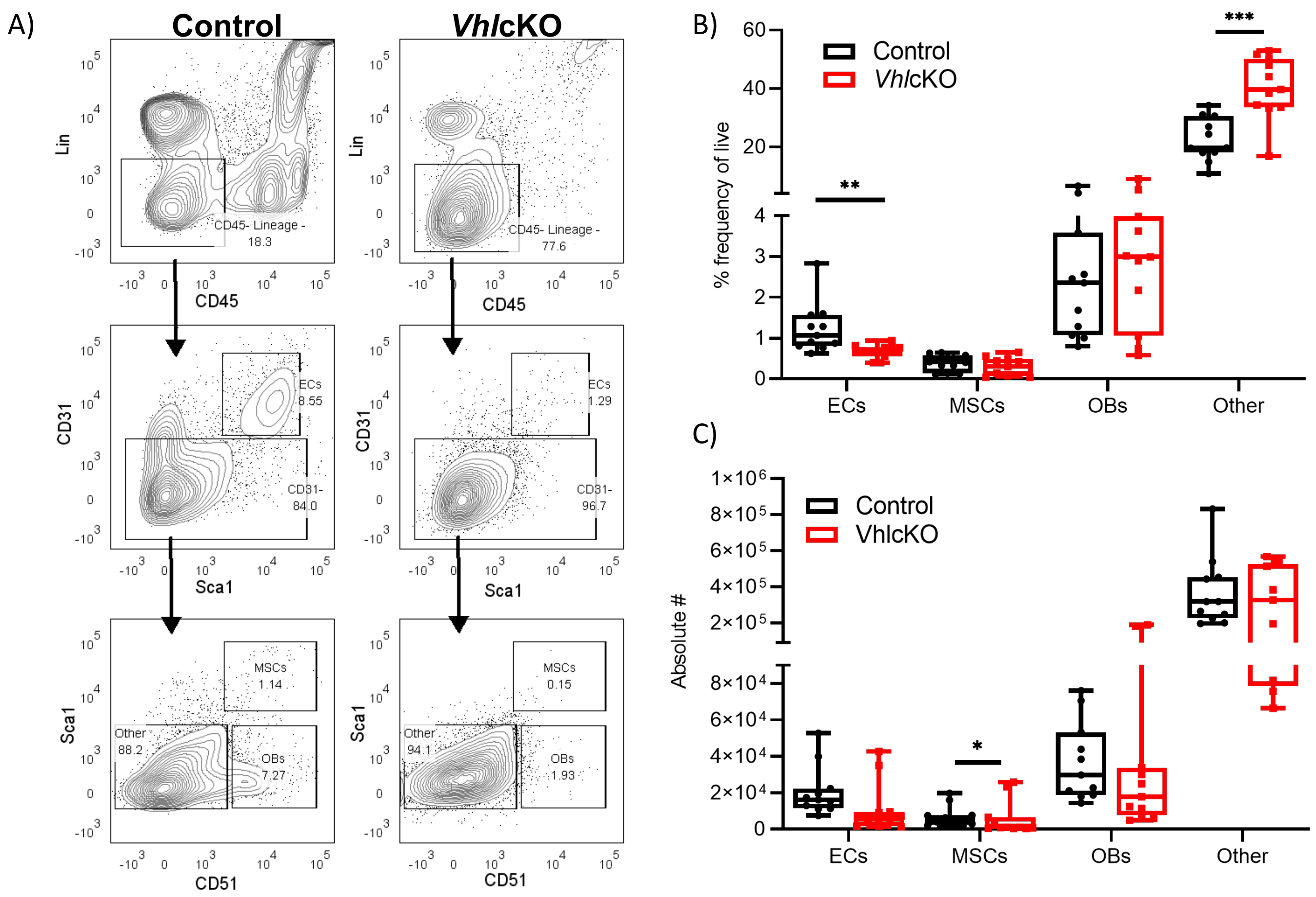

Supplemental Figure 6. Flow cytometric analysis of *Vhl*cKO bones. A) Flow cytometry gating strategy for bone niche cells (ECs, MSCs, OBs) after 2hr bone digest; B) frequencies and C) absolute numbers of each cell subset out of the live cell gate (not shown). $p<0.05$*, $p<0.01$**, $p<0.001$*** two-tailed Student's t-test.

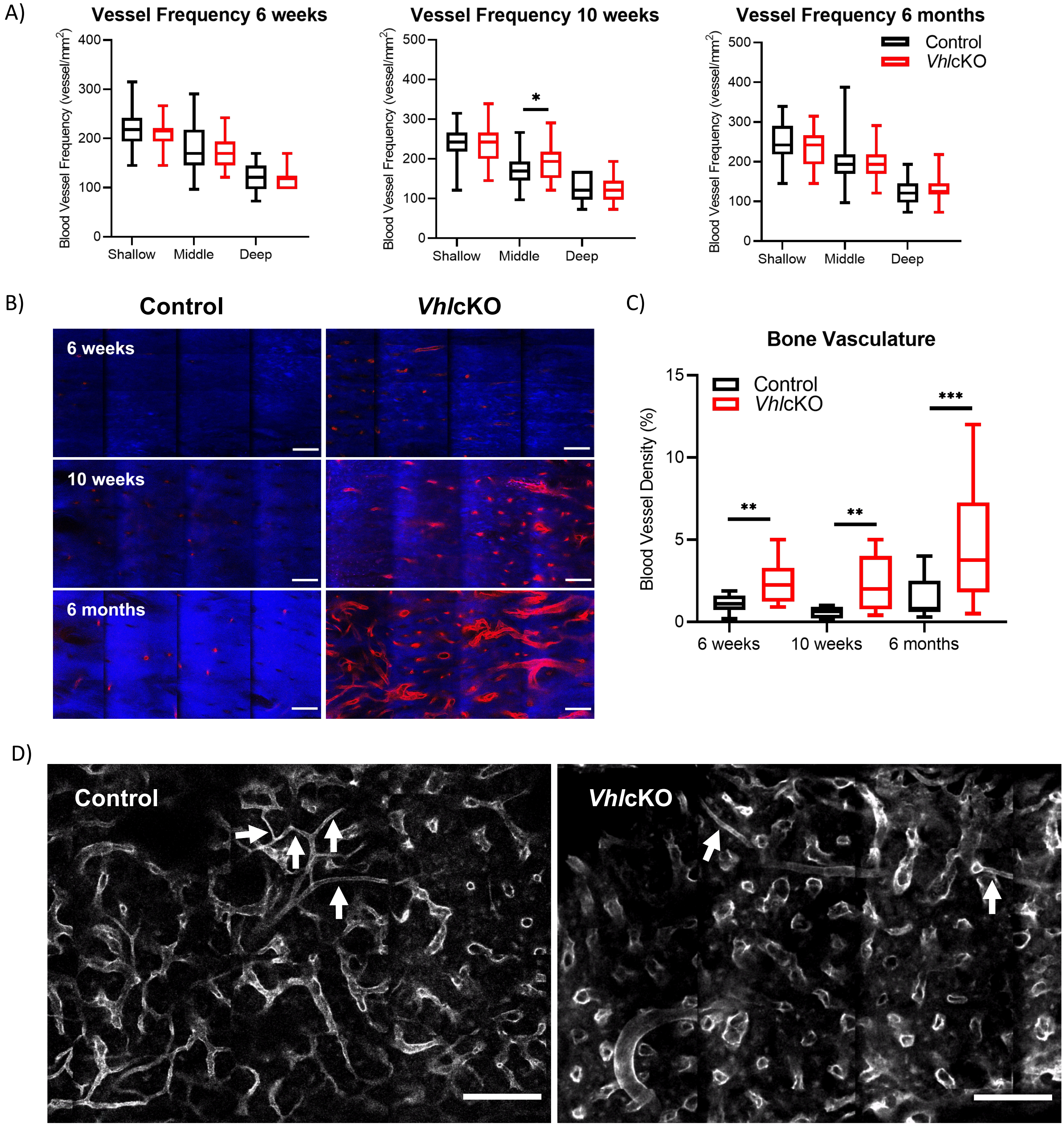

Supplemental Figure 7. Ex vivo imaging of bone and bone marrow vasculature in *Vhl*cKO and control mice. A) Quantification of age dependent vessel frequency in the diaphyseal BM of uDISCO cleared femurs at different depths (0-30 µm (shallow BM), 75-105 µm (middle BM), and 150-180 µm (deep BM) below the endosteum; B) maximum intensity projection images of cortical bone vascularization and C) quantification of cortical vessel density. Red: blood vessels (labeled with Alexa647 conjugated antibodies against CD31, CD144, and Sca-1), Blue: bone (SHG); D) maximum intensity projections of BM blood vessels within 50 µm of the endosteum in femurs of control (left) and *Vhl*cKO (right) mice; Grayscale: blood vessels (labeled with Alexa647 conjugated antibodies against CD31, CD144, and Sca-1). White arrows point to small diameter arterioles. Scale bars ~100 μm. *p<0.05, **p<0.01,***p<0.001, two-tailed Student's t-test.

Supplemental Table 1. List of the fluorochrome-labeled monoclonal antibodies used for flow cytometry and vessel staining sorted by experimental cocktail

Cocktail	Antigen	Clone	Fluorochrome	Source
Lineage Stain	CD45	30F11	FITC	Biolegend
	CD19	6D5	PE	Biolegend
	Gr1	RB6-8C5	PE-Cy7	Biolegend
	CD3	145-2C11	APC	Biolegend
	CD11b	M1/70	APC-Cy7	Biolegend
	Propidium iodide (PI)		Viability Dye	Sigma-Aldrich
Hematopoietic Progenitor	CD3	145-2C11	biotin	Biolegend
	CD4	Gk1.5	biotin	Biolegend
	CD8	53.6.7	biotin	Biolegend
	CD11b	M1/70	biotin	Biolegend
	CD19	6D5	biotin	Biolegend
	Nk1.1	PK136	biotin	Biolegend
	Ter119	TER119	biotin	Biolegend
	Gr1	RB6-8C5	biotin	Biolegend
	CD150	mShad150	FITC	eBioscience
	CD135 (Flk2)	A2F10	PE	eBioscience
	CD48	HM48-1	PE-Cy7	Biolegend
	CD127	IL-7Ra	APC	Biolegend
	CD117 (cKit)	2B8	APC-Cy7	Biolegend
	Sca1 (Ly-6A/E)	D7	BV510	Biolegend
	CD45	30-F11	PerCP Cy5.5	Biolegend
	Streptavidin	-	Pacific Blue	Life Technologies
	DAPI (4′,6-diamidino-2-phenylindole)		Viability Dye	Sigma-Aldrich
B cell Development	CD19	6D5	FITC	Biolegend
	CD43	1B11	PE	Biolegend
	B220	RA3-6B2	PE-Cy7	Biolegend
	IgD	12-26c.2a	BV510	Biolegend
	IgM	RMM-1	BV421	Biolegend
	CD117 (cKit)	2B8	APC-Cy7	Biolegend
	Propidium iodide (PI)		Viability Dye	Sigma-Aldrich
Transplant Lineage Stain	CD19	6D5	PE	Biolegend
	Gr1	RB6-8C5	PE-Cy7	Biolegend
	CD3	145-2C11	APC	Biolegend
	CD11b	M1/70	biotin	Biolegend
	Streptavidin	-	Pacific Blue	Life Technologies
	CD45.1	A20	FITC	Biolegend
	CD45.2	104	APC-Cy7	Biolegend
	Propidium iodide (PI)		Viability Dye	Sigma-Aldrich
Trasplant B cell development	CD19	6D5	FITC	Biolegend
	CD43	1B11	PE	Biolegend
	B220	RA3-6B2	PE-Cy7	Biolegend
	IgD	12-26c.2a	BV510	Biolegend
	IgM	RMM-1	BV421	Biolegend
	CD117 (cKit)	2B8	APC-Cy7	Biolegend
	CD45.1	A20	BUV395	BD Horizon
	CD45.2	104	APC	Biolegend
	Propidium iodide (PI)		Viability Dye	Sigma-Aldrich
Apoptosis in B cell development	CD43	1B11	PE	Biolegend
	B220	RA3-6B2	PE-Cy7	Biolegend
	IgD	12-26c.2a	BV510	Biolegend
	IgM	RMM-1	BV421	Biolegend
	CD117 (cKit)	2B8	APC-Cy7	Biolegend
	CD19	6D5	APC	Biolegend
	CD45	30-F11	BUV395	BD Horizon
	7AAD	-	7AAD	Biolegend
	Annexin V	-	FITC	Biolegend

	IgG1, κ (Isotype Control)	MOPC-21	FITC	Biolegend
Proliferation Stain Fractions A-C	CD19	6D5	FITC	Biolegend
	CD43	1B11	PE	Biolegend
	B220	RA3-6B2	PE-Cy7	Biolegend
	CD117 (cKit)	2B8	APC-Cy7	Biolegend
	CD45	30-F11	PerCP Cy5.5	Biolegend
	Ki67	16A8	APC	Biolegend
	IgG2a,k (Isotype Control)	RTK2758	APC	Biolegend
	DAPI (4′,6-diamidino-2-phenylindole)		Viability Dye	Sigma-Aldrich
Proliferation Stain Fractions D-F	IgD	11-26c.2a	FITC	Biolegend
	CD43	1B11	PE	Biolegend
	IgM	RMM-1	PE-Cy7	Biolegend
	B220	RA3-6B2	APC-Cy7	Biolegend
	CD45	30-F11	PerCP Cy5.5	Biolegend
	Ki67	16A8	APC	Biolegend
	IgG2a,k (Isotype Control)	RTK2758	APC	Biolegend
	DAPI (4′,6-diamidino-2-phenylindole)		Viability Dye	Sigma-Aldrich
Pimonidazole analysis of LSKs	CD3	145-2C11	biotin	Biolegend
	CD4	Gk1.5	biotin	Biolegend
	CD8	53.6.7	biotin	Biolegend
	CD11b	M1/70	biotin	Biolegend
	CD19	6D5	biotin	Biolegend
	Nk1.1	PK136	biotin	Biolegend
	Ter119	TER119	biotin	Biolegend
	Gr1	RB6-8C5	biotin	Biolegend
	Streptavidin	-	Pacific Blue	Life Technologies
	CD45	30-F11	PE	eBioscience
	CD117 (cKit)	2B8	APC	Biolegend
	Sca1 (Ly-6A/E)	D7	BV510	Biolegend
	B220	RA3-6B2	PE-Cy7	Biolegend
	Fixable viability	-	e780	eBioscience
	anti-PIM	-	FITC	HypoxyProbe
	IgG1, κ (Isotype Control)	MOPC-21	FITC	Biolegend
Pimonidazole analysis of B cell development	CD19	6D5	PECy5	Biolegend
	CD43	1B11	PE	Biolegend
	B220	RA3-6B2	PE-Cy7	Biolegend
	IgD	12-26c.2a	BV510	Biolegend
	IgM	RMM-1	BV421	Biolegend
	CD117 (cKit)	2B8	APC-Cy7	Biolegend
	anti-PIM	-	FITC	HypoxyProbe
	IgG1, κ (Isotype Control)	MOPC-21	FITC	Biolegend
Imaging antibodies	CD144	BV13	Alexa647	Biolegend
	CD31	MEC13.3	Alexa647	Biolegend
	Sca1 (Ly-6A/E)	D7	Alexa647	Biolegend
Bone digest stain	CD3	145-2C11	PE-Cy7	Biolegend
	CD4	Gk1.5	PE-Cy7	Biolegend
	CD8	53.6.7	PE-Cy7	Biolegend
	CD11b	M1/70	PE-Cy7	Biolegend
	CD19	6D5	PE-Cy7	Biolegend
	Gr1	RB6-8C5	PE-Cy7	Biolegend
	Nk1.1	PK136	PE-Cy7	Biolegend
	Ter119	TER119	PE-Cy7	Biolegend
	CD45	30F11	BV421	Biolegend
	CD31	390	APC	Biolegend
	Sca1 (Ly-6A/E)	D7	BV510	Biolegend
	CD51	RMV-7	Biotin	Biolegend
	IgG1,k (Isotype Control)	RTK2071	Biotin	Biolegend
	Streptavidin	-	PE	eBioscience
	Propidium iodide (PI)		Viability Dye	Sigma-Aldrich
